# Multi-Omics and Functional Analysis of *Salmonella* ArtAB Toxin Activity Reveals Transcriptomic Remodeling and Epithelial Barrier Dysfunction

**DOI:** 10.3390/toxins18090397

**Published:** 2026-09-17

**Authors:** Noah M. Souza, Alexander O. Hale, Richard S. Beard, Juliette K. Tinker

**Affiliations:** 1 Biomolecular Sciences Graduate Programs, Boise State University, Boise, ID 83725, USA; noahsouza@u.boisestate.edu; 2 Department of Biological Sciences, Boise State University, Boise, ID 83725, USA; wofw.alex1@gmail.com; 3 Department of Molecular Pharmacology and Physiology, Morsani College of Medicine, University of South Florida, Tampa, FL 33612, USA; rbeard@usf.edu

**Keywords:** AB_5_ toxin, ArtAB, *Salmonella* Typhimurium, transcriptomics, proteomics, epithelial barrier

## Abstract

AB_5_ toxins are important virulence factors produced by Gram-negative bacteria and have been identified in many non-typhoidal *Salmonella* (NTS) serovars; however, their effects on host cell signaling and epithelial barrier function remain poorly understood. This study characterized the molecular and functional effects of purified ArtAB toxin and its binding subunit, ArtB, from multidrug-resistant *Salmonella* Typhimurium definitive type 104 (DT104). Equimolar concentrations of ArtAB (10 µg/mL) and ArtB (7.25 µg/mL) were evaluated in Chinese hamster ovary (CHO-K1) cells using cytotoxicity assays, quantitative proteomics, and mRNA sequencing, while barrier function was assessed in C2BBe1 human intestinal epithelial cells using electric cell-substrate impedance sensing (ECIS). In CHO-K1 cells, both proteins exhibited minimal cell death. ArtB elicited pronounced proteomic changes associated with endoplasmic reticulum (ER) homeostasis, vesicular trafficking, and lysosomal processing, but limited transcriptional responses. ArtAB instead altered host transcriptional programs associated with cell growth, inflammatory signaling, and cytoskeletal organization. Unlike ArtB, ArtAB significantly reduced barrier function in C2BBe1 cells. Together, these findings show that ArtAB remodels host cell signaling without overt cytotoxicity in CHO-K1 cells and produces a distinct functional effect in C2BBe1 cells. Although obtained in different cellular models, these results provide insight into the potential role of ArtAB in NTS pathogenesis.

## 1. Introduction

Non-typhoidal *Salmonella* (NTS) is a major global public health concern due to increasing antibiotic resistance and significant disease burden [1]. Despite improved surveillance, multidrug-resistant isolates continue to emerge, increasing the likelihood of severe invasive disease with high mortality [2,3]. The majority of human NTS infections are foodborne and linked to contaminated animal sources, such as cattle, poultry, and swine [4,5]. NTS infections are usually limited to gastroenteritis and remain localized in the intestine, although invasive cases resulting in bacteremia and sepsis can occur, particularly in populations with high rates of comorbidities [6]. In contrast to the broad animal reservoirs associated with NTS, typhoidal *Salmonella* (TS) is restricted to humans and commonly causes invasive systemic disease following fecal-oral transmission [7].

The burden of NTS is substantial from both healthcare and agricultural perspectives. In 2023, NTS was considered the most expensive foodborne pathogen in the U.S., accounting for an estimated total cost of $17 billion and a mean per-case cost of ~$16,500 due to medical expenses, lost productivity, and premature deaths [8,9]. With over 2600 identified serovars within *Salmonella enterica* and numerous virulence factors contributing to infection and transmission in both humans and animals, the pathogen is often considered from a One Health perspective [10,11]. Although host-specific vaccines have been developed against major serovars for agriculturally important animals, NTS continues to adapt and persist [12,13,14].

Decades of research have focused on understanding NTS virulence mechanisms that contribute to human disease, including toxins, capsules, flagella, fimbriae, and secretion systems. These virulence factors contribute to host colonization, intestinal invasion, and immune evasion during infection [15]. However, the virulence mechanisms of NTS and TS differ and include distinct Vi capsule production and immune suppression strategies [7,16]. Among the virulence factors associated with TS is typhoid toxin (TT), an A_2_B_5_ toxin that is expressed within host cells and triggers symptoms of typhoid fever in laboratory animals [17]. In contrast, considerably less is known about the cellular effects of related AB_5_ toxins produced by NTS.

AB_5_ toxins are a family of multi-subunit proteins produced by several Gram-negative bacteria, in which the pentameric B-subunit facilitates host cell binding and endocytosis, and the enzymatically active A-subunit modulates host cell function [18]. Prime examples include cholera toxin (CT) from *Vibrio cholerae*, pertussis toxin (PT) from *Bordetella pertussis*, and heat-labile toxin (LT) from *E. coli*. Another member of this family is ArtAB, an AB_5_ toxin identified in NTS. While ArtAB is thought to share common ancestry with TT and its alternate B-subunit, PltC, its effects on mammalian host cell signaling and epithelial barrier function remain poorly characterized [19,20,21]. TT has been shown to disrupt tight junctions in the blood-brain barrier, highlighting the need to determine whether ArtAB similarly affects epithelial barrier function during NTS infection [22]. Defining these cellular responses may help clarify the contribution of ArtAB to NTS pathogenesis.

ArtAB was first identified in 2005 in the highly virulent strain *S.* Typhimurium definitive type 104 (DT104) [23]. The *artAB* genes are encoded within a Gifsy-1-like prophage, and this toxin has been shown to be induced by mitomycin C, possess adenosine diphosphate (ADP)-ribosyltransferase activity, and increase intracellular cyclic adenosine monophosphate (cAMP) [23,24,25]. Similar to TT, ArtAB can also be expressed by *Salmonella* within macrophages [26]. We previously cloned, expressed, and purified ArtAB (~93.1 kDa) and its B-subunit, ArtB (~67.5 kDa), demonstrating distinct cellular phenotypes and limited cytotoxicity following treatment in Chinese hamster ovary (CHO-K1) cells [27]. However, the downstream molecular consequences of ArtAB intoxication on host signaling remained unclear. CHO-K1 cells were selected for multi-omics characterization based on their established use for evaluating the cellular effects of many AB_5_ toxins, such as CT and PT [28,29,30,31,32]. Our molecular findings in CHO-K1 cells motivated subsequent studies in C2BBe1 cells, a human intestinal epithelial cell line, to evaluate barrier function. We hypothesized that ArtAB would induce host cellular responses distinct from those elicited by its binding subunit, ArtB, and alter intestinal epithelial barrier integrity. This study aimed to characterize specific host cellular responses to ArtAB using proteomics and transcriptomics in CHO-K1 cells and to evaluate epithelial barrier effects in C2BBe1 cells. To our knowledge, this study provides the first integrated multi-omics characterization of ArtAB and identifies distinct molecular and functional responses across complementary cellular models.

## 2. Results

### 2.1. ArtAB Induces Minimal Cytotoxicity in CHO-K1 Cells

**Figure 1:**
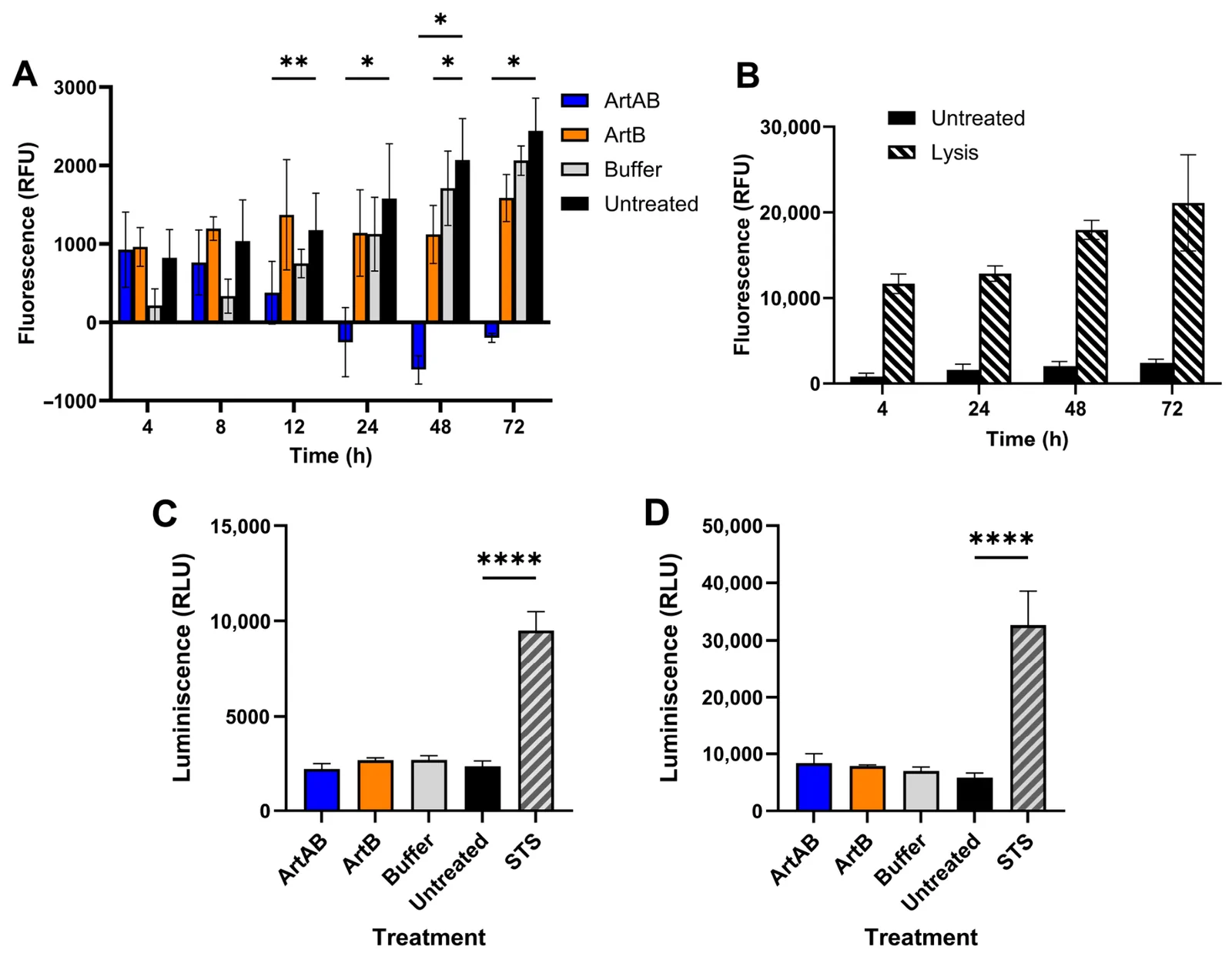
Cytotoxicity in CHO-K1 cells treated with 10 µg/mL ArtAB or 7.25 µg/mL ArtB. (**A**,**B**) CellTox Green cytotoxicity analysis following (**A**) treatment with ArtAB, ArtB, an equivalent volume of Buffer, or untreated cells, and (**B**) untreated and lysis solution-treated cells as negative and positive controls, respectively. Data are background-subtracted and presented as the mean ± SD from three independent experiments (*n* = 3 per treatment). Panel (**A**) was analyzed by two-way repeated-measures ANOVA with Dunnett’s multiple-comparisons test against untreated cells at each time point (* *p* ≤ 0.05 and ** *p* ≤ 0.01); panel B was not statistically analyzed. (**C**,**D**) Caspase-Glo 3/7 apoptosis analysis following treatment with ArtAB, ArtB, an equivalent volume of Buffer, or untreated cells for (**C**) 4 h or (**D**) 24 h. 5 µM staurosporine (STS) was added 4 h before each measurement as a positive control. Data are background-subtracted and presented as the mean ± SD from one representative experiment (*n* = 4 technical replicates per treatment). Panels (**C**,**D**) were analyzed by one-way ANOVA with Dunnett’s multiple-comparisons test against untreated cells (**** *p* ≤ 0.0001).

Purified ArtAB at 10 µg/mL was previously identified as the minimum concentration shown to induce morphological changes and limited cytotoxicity in CHO-K1 cells [27]. While the previous study evaluated cell viability using crystal violet staining and an alamarBlue metabolic activity assay, the present study used complementary assays to assess membrane integrity and apoptotic activity. Thus, CHO-K1 cells were treated with equimolar concentrations of ArtAB (10 µg/mL) or ArtB (7.25 µg/mL), or an equivalent volume of Buffer (1× PBS + 5% glycerol) to evaluate cell lytic and apoptotic effects (Figure 1). Cell lysis was assessed using CellTox Green fluorescence (Figure 1A,B), while apoptosis was measured with Caspase-Glo 3/7 luminescence (Figure 1C,D). Kit-supplied lysis solution and 5 µM staurosporine (STS) were used as positive controls for cell lysis and apoptosis, respectively. For both assays, higher values indicate greater cell death.

Over the 72 h time course, ArtAB induced minimal cell lysis, with fluorescence values remaining comparable to untreated cells at 4 and 8 h. Fluorescence was significantly lower in ArtAB-treated cells than in untreated cells at 12, 24, 48, and 72 h (*p* = 0.0062, 0.0125, 0.0129, and 0.0180, respectively), indicating no increase in cytotoxicity at these time points. ArtB produced slightly higher fluorescence than ArtAB across the entire time course, but only significantly differed from untreated cells at 48 h (*p* = 0.0195) (Figure 1A). As expected, fully lysed cells showed substantially greater fluorescence than untreated cells, confirming the performance of the positive control (Figure 1B). Consistent with these cytotoxicity findings, ArtAB and ArtB did not induce significant apoptosis at either 4 or 24 h, with values similar to Buffer and untreated cells (Figure 1C,D). In contrast, STS produced a robust increase in Caspase-Glo 3/7 activity at both time points (*p* < 0.0001), confirming assay performance.

### 2.2. Proteomic Analysis of CHO-K1 Cells Identifies ArtB-Associated Intracellular Processing and ArtAB-Associated Redox and Transcriptional Remodeling

**Figure 2:**
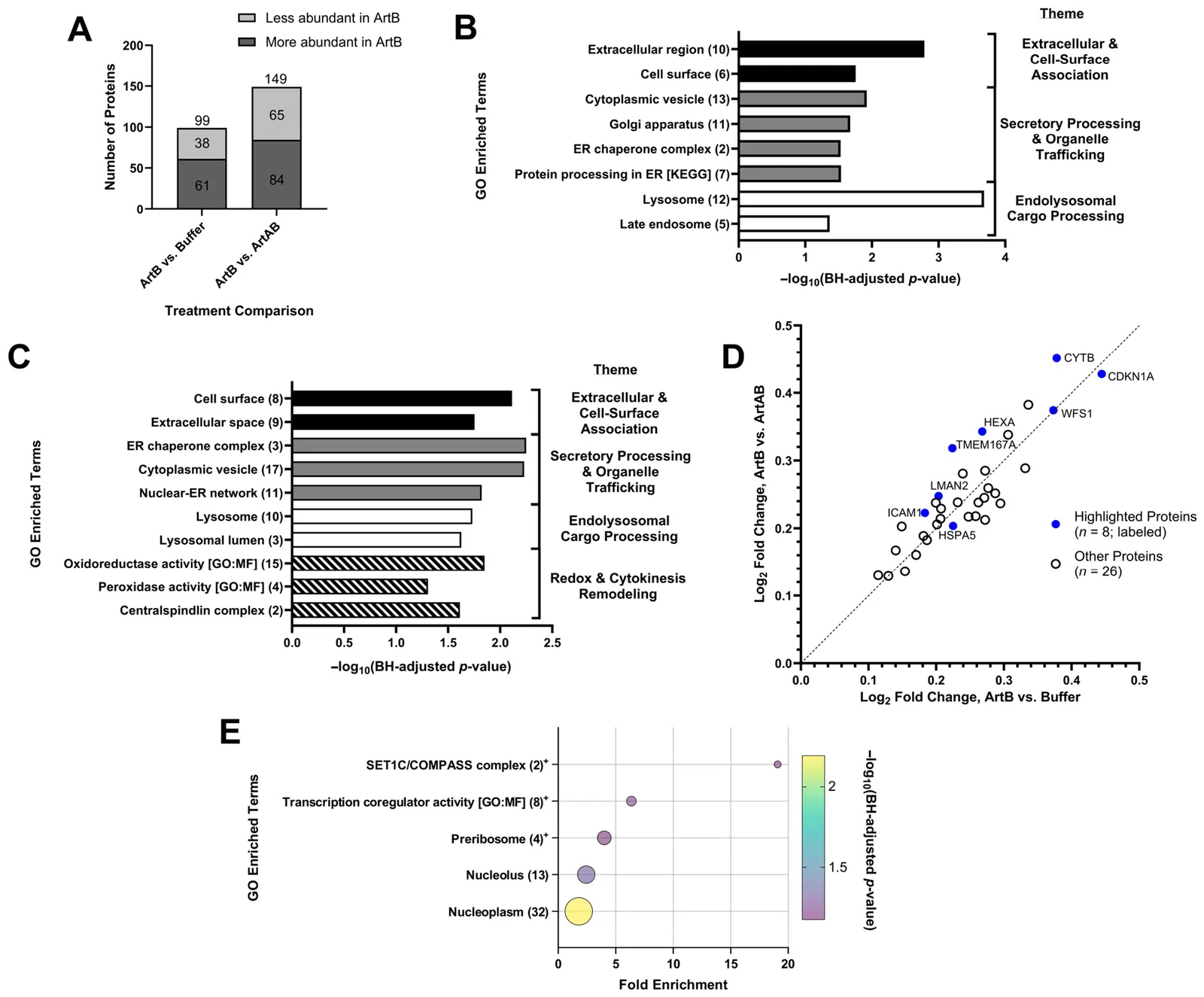
Proteomic analysis of ArtAB- and ArtB-treated CHO-K1 cells. Cells were treated with 10 µg/mL ArtAB, 7.25 µg/mL ArtB, or an equivalent volume of Buffer for 4 h. (**A**) Total numbers of differentially abundant proteins (DAPs) in ArtB vs. Buffer and ArtB vs. ArtAB, categorized as lower or higher in ArtB. (**B**,**C**) Representative terms significantly enriched among DAPs higher in ArtB relative to (**B**) Buffer or (**C**) ArtAB. Terms in both panels are grouped by biological theme and represent Gene Ontology cellular component (GO:CC) terms unless otherwise indicated in brackets. In panel (**C**), “Nuclear outer membrane-ER membrane network” was shortened to “Nuclear-ER network”. (**D**) Log_2_ fold changes for 34 DAPs that were more abundant in ArtB in both comparisons. Eight highlighted proteins are labeled, and the dashed line indicates equal fold changes between comparisons. (**E**) Representative terms significantly enriched among DAPs lower in ArtB relative to ArtAB, ordered by decreasing fold enrichment. Point color represents −log_10_(Benjamini–Hochberg (BH)-adjusted *p*-value) and point size represents the number of proteins mapped to each term. In panels (**B**,**C**,**E**) numbers in parentheses indicate mapped protein counts. A plus sign (+) denotes terms meeting the exploratory functional enrichment BH-adjusted *p*-value threshold of 0.10; unmarked terms met the primary threshold of 0.05.

Based on our previous observations of ArtAB-associated cellular effects with minimal cytotoxicity, a 4 h exposure was selected to characterize early molecular responses in CHO-K1 cells before the morphological changes observed at 24 h [27]. As in Section 2.1, cells were treated with 10 µg/mL ArtAB, 7.25 µg/mL ArtB, or an equivalent volume of Buffer for 4 h, after which proteins were extracted and analyzed by LC-MS/MS. Differential protein abundance was assessed for the ArtAB vs. Buffer, ArtB vs. Buffer, and ArtB vs. ArtAB comparisons based on log_2_ fold change, and the resulting differentially abundant proteins (DAPs) were subjected to functional enrichment analysis (Figure 2).

The ArtB vs. Buffer (*n* = 99) and ArtB vs. ArtAB (*n* = 149) comparisons produced the largest numbers of DAPs (Figure 2A). In contrast, only one protein, Fos-related antigen 1 (FOSL1), was differentially abundant in the ArtAB vs. Buffer comparison; complete differential abundance data, including protein symbols, for all comparisons are provided in Table S1. All DAPs higher in ArtB relative to Buffer (*n* = 61) or ArtAB (*n* = 84) were subjected to functional enrichment analysis, which included Gene Ontology (GO) and Kyoto Encyclopedia of Genes and Genomes (KEGG). The enrichment results consisted primarily of GO cellular component (GO:CC) terms, and the complete enrichment results for both comparisons are provided in Table S2. Although some of the specific enriched terms differed between comparisons, representative GO:CC terms were organized into three shared biological themes reflecting intracellular processing. These included: (i) extracellular and cell-surface association, (ii) secretory processing and organelle trafficking, and (iii) endolysosomal cargo processing (Figure 2B,C). Among proteins more abundant in ArtB relative to Buffer, protein processing in the endoplasmic reticulum (ER) was identified as the only enriched KEGG pathway, while lysosome (GO:CC) was the most statistically significant term with a −log_10_(Benjamini–Hochberg (BH)-adjusted *p*-value) of 3.683 (Figure 2B). For proteins more abundant in ArtB relative to ArtAB, additional significant GO molecular function (GO:MF) terms mapped to a fourth theme associated with redox activity and cytokinesis remodeling (Figure 2C).

Since ArtB was the common treatment in both comparisons, the 34 DAPs higher in both ArtB vs. Buffer and ArtB vs. ArtAB were plotted against each other using their log_2_ fold changes (Figure 2D). Eight proteins were highlighted based on their concordance between comparisons, biological relevance, and representation of the enriched functional themes. These included ER-homeostasis proteins (HSPA5 and WFS1), trafficking-associated proteins (LMAN2 and TMEM167A), a lysosomal enzyme (HEXA), a cell-surface protein (ICAM1), and a cell-state protein (CDKN1A), and a metabolism-associated protein (CYTB). To further characterize the effects of ArtAB on the proteome, the 65 DAPs lower in ArtB relative to ArtAB were also subjected to functional enrichment analysis (Table S2). These proteins were primarily associated with transcriptional complexes, co-regulator activity, and the nucleoplasm (Figure 2E).

### 2.3. mRNA Sequencing Identifies ArtAB-Associated Changes in Cell Growth, Gene Regulation, and Immune-Related Pathways in CHO-K1 Cells

**Figure 3:**
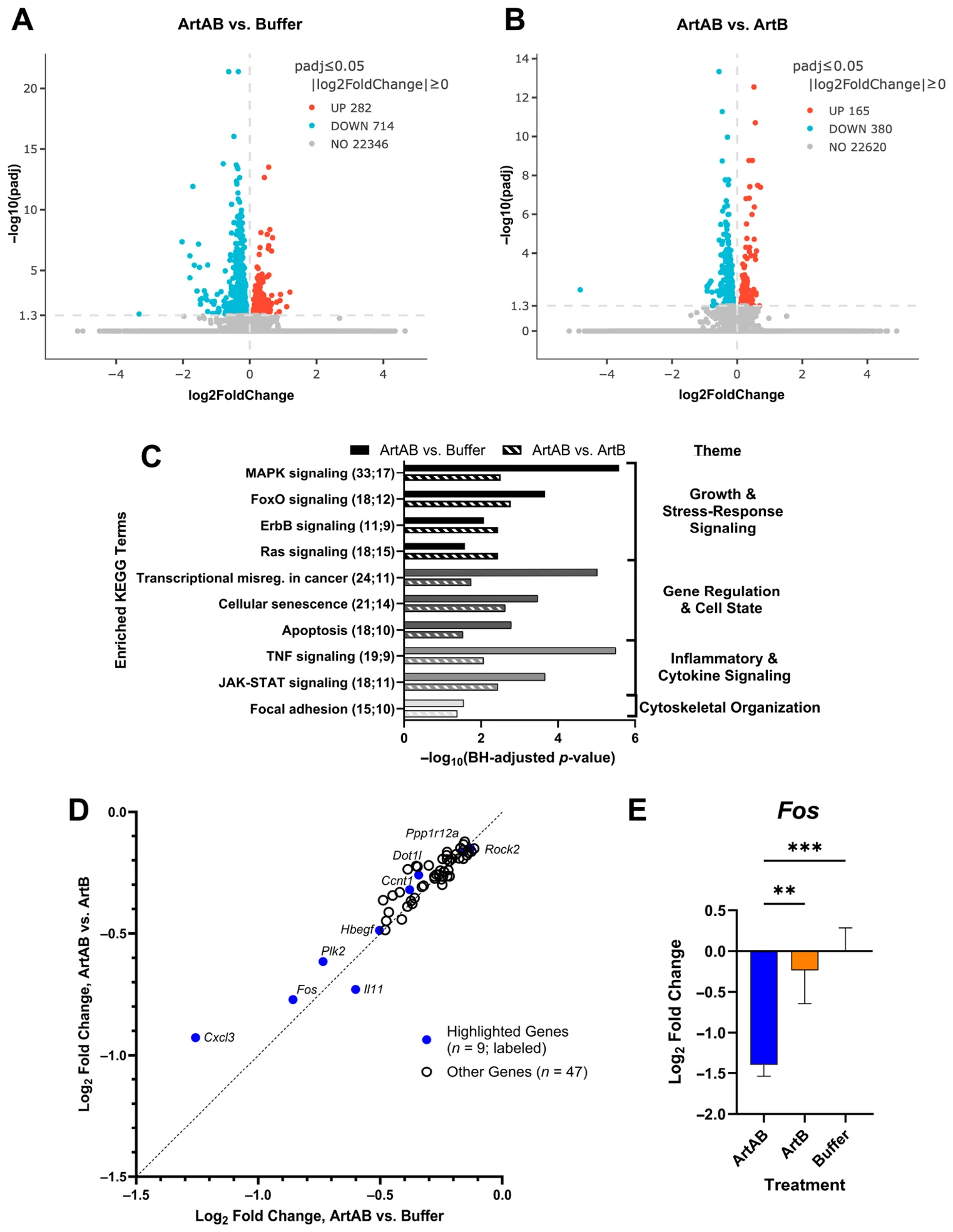
Transcriptomic analysis of ArtAB- and ArtB-treated CHO-K1 cells. Cells were treated with 10 µg/mL ArtAB, 7.25 µg/mL ArtB, or an equivalent volume of Buffer for 4 h. (**A**,**B**) Volcano plots of differential gene expression in (**A**) ArtAB vs. Buffer and (**B**) ArtAB vs. ArtB. (**C**) Representative KEGG pathways significantly enriched among differentially expressed genes (DEGs) downregulated in ArtAB relative to both Buffer and ArtB. Pathways were grouped by biological theme and ordered within each theme by decreasing −log_10_(Benjamini–Hochberg (BH)-adjusted *p*-value) for ArtAB vs. Buffer. Parentheses indicate mapped gene counts for ArtAB vs. Buffer and ArtAB vs. ArtB, separated by a semicolon. (**D**) Log_2_ fold changes for 56 DEGs downregulated in both comparisons that mapped to the pathways in panel (**C**). Highlighted genes are labeled, and the dashed line indicates equal fold changes between comparisons. (**E**) Relative *Fos* expression at 4 h measured by qRT-PCR. Data are presented as the mean ± SD (*n* = 4 per treatment) and were analyzed by one-way ANOVA with Tukey’s multiple-comparisons test (** *p* ≤ 0.01 and *** *p* ≤ 0.001).

Since the proteomic analysis did not reveal significant ArtAB-induced differential protein abundance at 4 h but did identify ArtAB-associated differences involving transcriptional complexes and the nucleoplasm, gene expression was next evaluated. CHO-K1 cells were treated under identical conditions for 4 h with 10 µg/mL ArtAB, 7.25 µg/mL ArtB, or an equivalent volume of Buffer, and total RNA was isolated for mRNA sequencing. Differential gene expression was assessed for the ArtAB vs. Buffer, ArtAB vs. ArtB, and ArtB vs. Buffer comparisons (Figure 3).

The ArtAB vs. Buffer (*n* = 996) and ArtAB vs. ArtB (*n* = 545) comparisons produced the largest numbers of differentially expressed genes (DEGs), as illustrated by the corresponding volcano plots (Figure 3A,B). In contrast, the ArtB vs. Buffer comparison yielded only 41 DEGs (Figure S1). Complete DEG results, including gene symbols, for all comparisons are provided in Table S3.

ArtAB vs. Buffer and ArtAB vs. ArtB yielded 714 and 380 downregulated genes, respectively (Figure 3A,B), which were subjected to KEGG enrichment analysis. The complete functional enrichment results for both comparisons are provided in Table S4. A substantial overlap in enriched pathways was observed between the two comparisons, and ten representative pathways were organized into biologically relevant themes to facilitate interpretation (Figure 3C). These themes included: (i) growth and stress response signaling, (ii) gene regulation and cell state, (iii) inflammatory and cytokine signaling, and (iv) cytoskeletal organization.

A total of 56 downregulated genes were shared between the ArtAB vs. Buffer and ArtAB vs. ArtB comparisons across the ten representative pathways, and their log_2_ fold changes were plotted against each other (Figure 3D). Nine genes were highlighted based on their concordance between comparisons, biological relevance, and representation of the enriched functional themes. These included growth signaling genes (*Fos*, *Hbegf*, and *Plk2*), regulatory genes (*Ccnt1* and *Dot1l*), inflammatory genes (*Cxcl3* and *Il11*), and cytoskeleton-associated genes (*Ppp1r12a* and *Rock2*). Mitogen-activated protein kinase (MAPK) signaling was among the most significantly enriched pathways for ArtAB vs. Buffer and ArtAB vs. ArtB with a −log_10_(BH-adjusted *p*-value) of 5.588 and 2.512, respectively. Since *Fos* exhibited one of the largest decreases in expression across both comparisons, its expression was further evaluated by qRT-PCR (Figure 3E).

**Figure 4:**
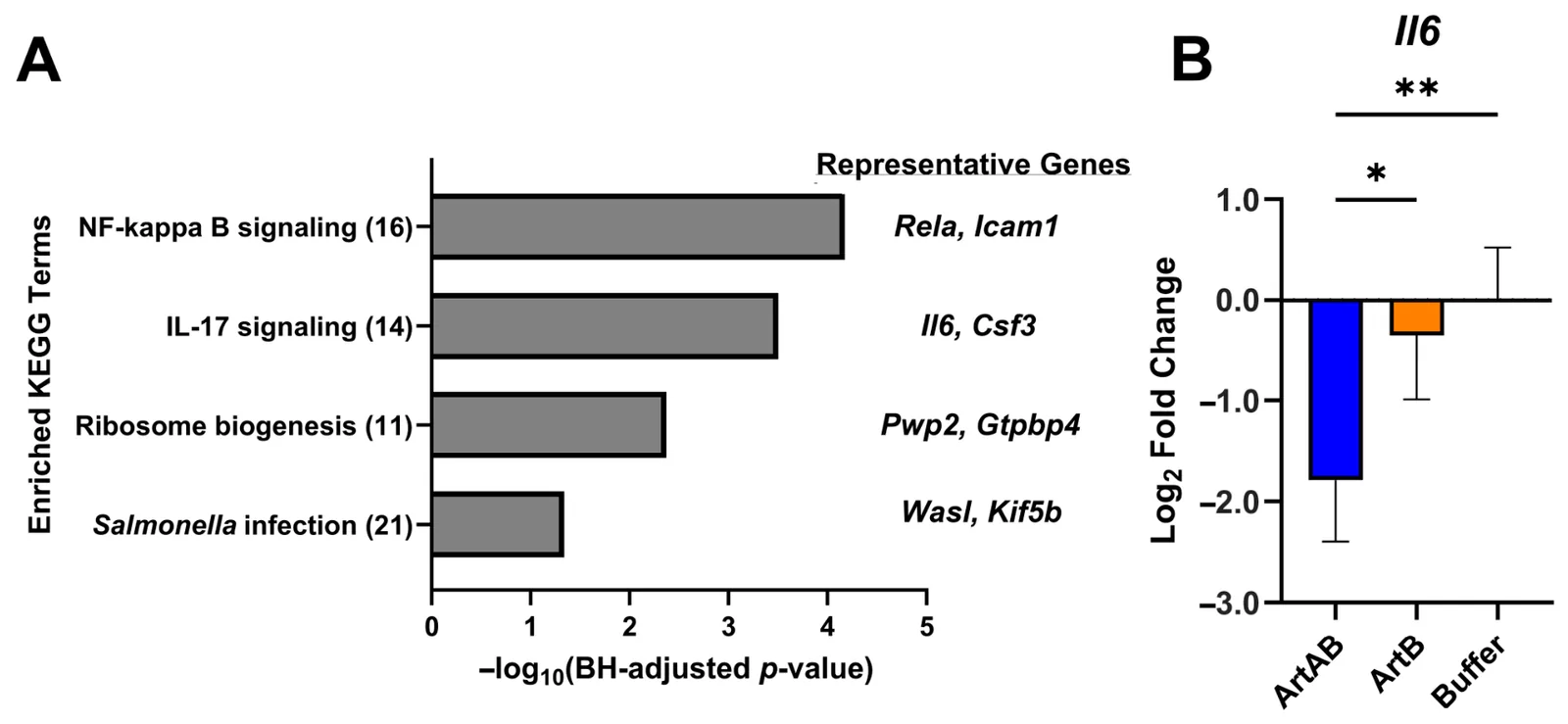
Comparison-specific transcriptional responses in CHO-K1 cells treated with ArtAB or ArtB. Cells were treated with 10 µg/mL ArtAB, 7.25 µg/mL ArtB, or an equivalent volume of Buffer for 4 h. (**A**) KEGG pathways significantly enriched among differentially expressed genes (DEGs) downregulated in ArtAB vs. Buffer that were not significantly enriched in ArtAB vs. ArtB. Numbers in parentheses indicate mapped gene counts, and representative genes are listed. (**B**) Relative *Il6* expression at 4 h measured by qRT-PCR. Data in panel (**B**) are presented as the mean ± SD (*n* = 4 per treatment) and were analyzed by one-way ANOVA with Tukey’s multiple-comparisons test (* *p* ≤ 0.05 and ** *p* ≤ 0.01).

Although Figure 3C highlights the major pathways shared across the two ArtAB comparisons for downregulated genes, additional KEGG pathways were significantly enriched among genes downregulated in ArtAB vs. Buffer but not in ArtAB vs. ArtB (Figure 4).

These ArtAB vs. Buffer-specific KEGG pathways included nuclear factor kappa B (NF-κB), interleukin-17 (IL-17), ribosome biogenesis, and *Salmonella* infection. *Il6* was among the most downregulated genes within these comparison-specific pathways, and its decreased expression following ArtAB treatment was further validated by qRT-PCR (Figure 4B).

### 2.4. Upregulated ArtAB-Associated Genes in CHO-K1 Cells Further Support Cytoskeletal Remodeling

**Figure 5:**
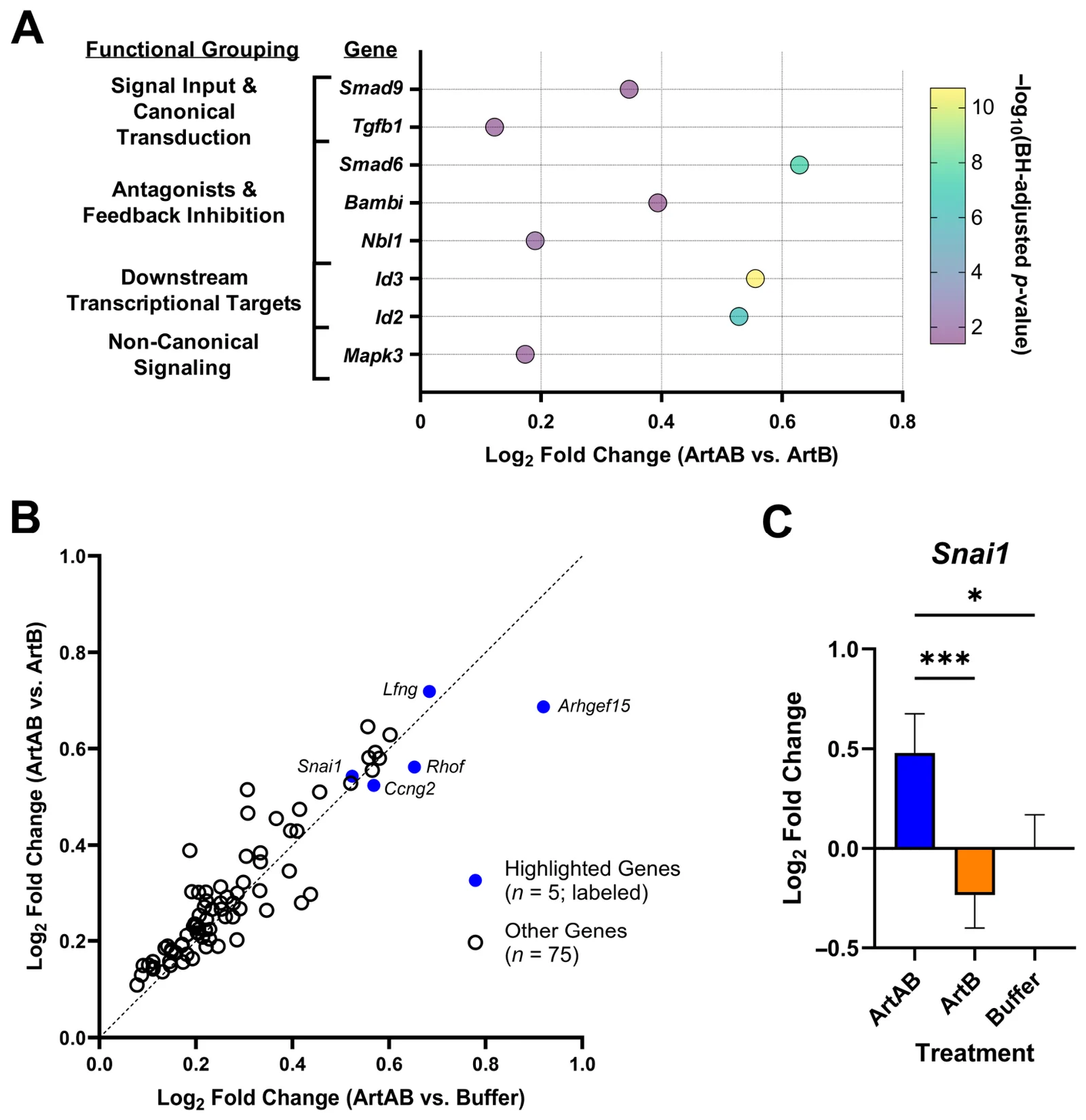
Upregulated ArtAB-associated transcriptional responses in CHO-K1 cells. Cells were treated with 10 µg/mL ArtAB, 7.25 µg/mL ArtB, or an equivalent volume of Buffer for 4 h. (**A**) Eight DEGs upregulated in ArtAB vs. ArtB that mapped to the TGF-β signaling pathway, grouped by functional role. Point color indicates −log_10_(Benjamini–Hochberg (BH)-adjusted *p*-value). (**B**) Log_2_ fold changes for 80 DEGs upregulated in ArtAB relative to both Buffer and ArtB. Highlighted genes are labeled, and the dashed line indicates equal fold changes between comparisons. (**C**) Relative *Snai1* expression at 4 h measured by qRT-PCR. Data in panel (**C**) are presented as the mean ± SD (*n* = 4 per treatment) and were analyzed by one-way ANOVA with Tukey’s multiple-comparisons test (* *p* ≤ 0.05 and *** *p* ≤ 0.001).

As shown in Figure 3A, 282 upregulated DEGs were identified in the ArtAB vs. Buffer comparison, but no KEGG pathways met the BH-adjusted *p*-value significance threshold. In contrast, functional enrichment analysis of the 165 genes upregulated in ArtAB relative to ArtB identified transforming growth factor-beta (TGF-β) signaling as the only significantly enriched KEGG pathway with a BH-adjusted *p*-value of 0.00337 (Table S4). Further analysis of upregulated ArtAB-associated responses focused on TGF-β signaling and genes shared across the two ArtAB comparisons (Figure 5).

Eight genes mapped to the TGF-β signaling pathway and were organized according to their predominant biological functions: (i) signal input and canonical transduction, (ii) antagonists and feedback inhibition, (iii) downstream transcriptional targets, and (iv) non-canonical signaling (Figure 5A). To further examine upregulated ArtAB-associated responses beyond TGF-β signaling, the 80 DEGs shared between the ArtAB vs. Buffer and ArtAB vs. ArtB comparisons were examined directly. Specifically, their log_2_ fold changes in each comparison were plotted against each other, and five genes were highlighted based on their magnitude and associations with TGF-β-related processes, including cell proliferation, migration, and cytoskeletal regulation (Figure 5B). These genes were *Snai1*, *Ccng2*, *Lfng*, *Rhof*, and *Arhgef15*. Since Overgaard et al. (2021) reported ArtAB-associated cytoskeletal rearrangements in CHO-K1 cells [27], *Snai1* upregulation was further evaluated and confirmed by qRT-PCR (Figure 5C).

### 2.5. ArtAB Decreases Epithelial Barrier Resistance in C2BBe1 Cells During Electric Cell-Substrate Impedance Sensing (ECIS)

**Figure 6:**
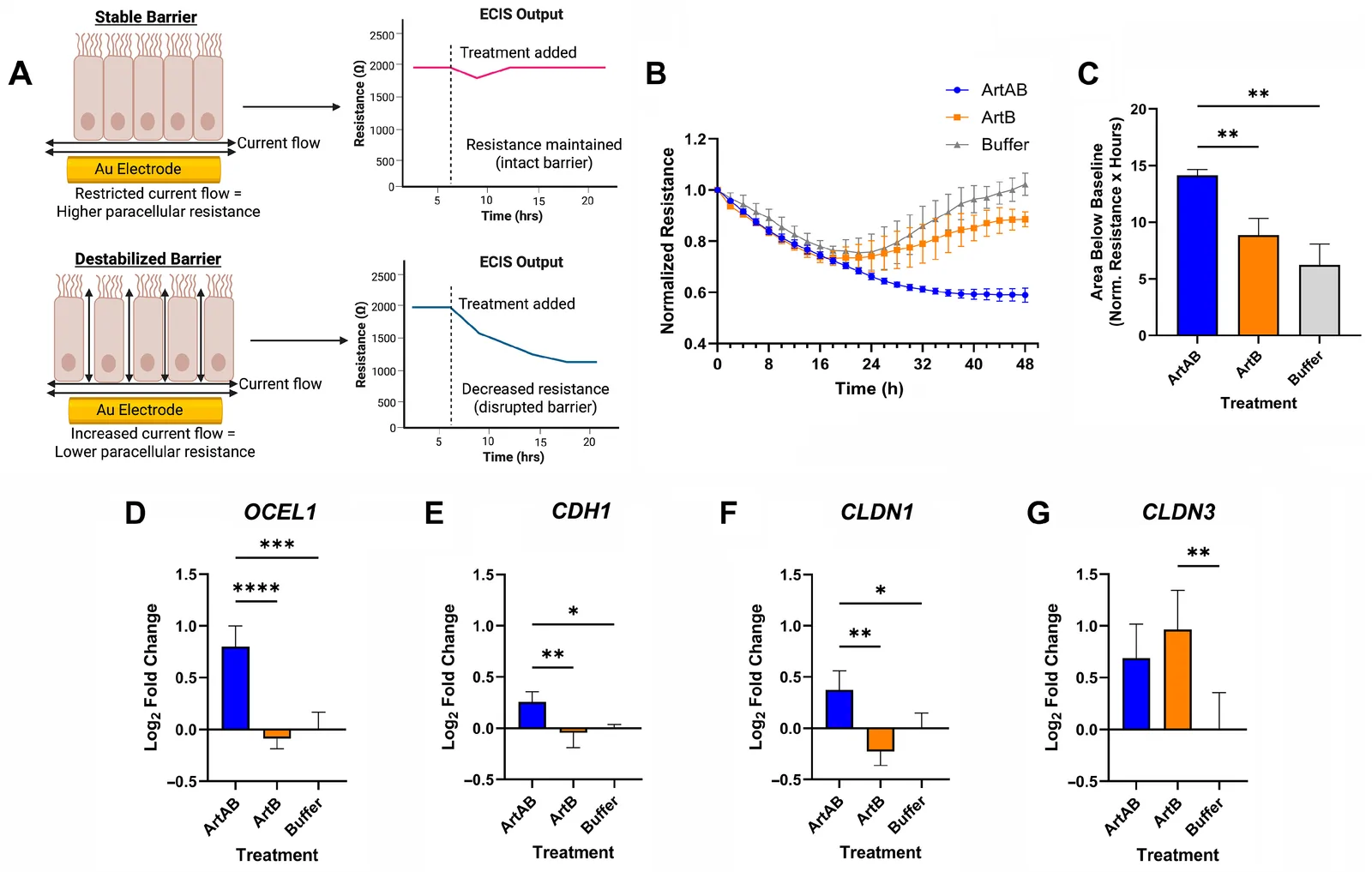
Effects of ArtAB and ArtB on C2BBe1 epithelial barrier resistance and junction-associated gene expression. Cells were treated with 10 µg/mL ArtAB, 7.25 µg/mL ArtB, or an equivalent volume of Buffer. (**A**) Schematic overview of electric cell-substrate impedance sensing (ECIS) resistance measurements in intact (top) and disrupted (bottom) epithelial monolayers (Created in BioRender. Souza, N. (2026) https://BioRender.com/5vk9eii). (**B**) Normalized resistance over 48 h, with the measurement nearest to each 2 h time point selected from the continuous ECIS recording for graphical presentation. (**C**) Area under the curve (AUC) below the baseline (*y* = 1) calculated from the complete continuous normalized resistance recordings within each independent experiment. Data in panels (**B**,**C**) are presented as the mean ± SD (*n* = 3 per treatment). (**D**–**G**) Relative expression of junction-associated genes measured by qRT-PCR at 20 h: (**D**) *OCEL1*, (**E**) *CDH1*, (**F**) *CLDN1*, and (**G**) *CLDN3*. Data are presented as the mean ± SD (*n* = 4 per treatment). Panels (**C**–**G**) were analyzed by one-way ANOVA with Tukey’s multiple-comparisons test (* *p* ≤ 0.05, ** *p* ≤ 0.01, *** *p* ≤ 0.001, **** *p* ≤ 0.0001).

Based on the ArtAB transcriptional data suggesting altered cytoskeletal organization in Figure 5, ECIS was used to evaluate toxin effects on epithelial barrier resistance. C2BBe1 cells, a clone of the polarized human intestinal epithelial Caco-2 cell line, were selected to promote the formation of functional epithelial barriers [33]. C2BBe1 cells were treated with 10 µg/mL ArtAB, 7.25 µg/mL ArtB, or an equivalent volume of Buffer, and paracellular resistance was continuously measured for 48 h (Figure 6). ECIS measures the resistance to an electrical current passing through gold electrodes beneath adherent cells, providing a real-time assessment of epithelial barrier integrity (Figure 6A).

During the first 15 h, normalized resistance in all treatment groups initially decreased to approximately 0.75 with no distinguishing differences. Between 15 and 20 h, the responses diverged, with resistance in ArtAB-treated cells continuing to decrease, whereas ArtB and Buffer started to recover (Figure 6B). At approximately 32 h, normalized resistance was 0.612 ± 0.012 for ArtAB, 0.791 ± 0.076 for ArtB, and 0.860 ± 0.077 for Buffer. At approximately 48 h, normalized resistance was 0.590 ± 0.027 for ArtAB, 0.885 ± 0.030 for ArtB, and 1.022 ± 0.044 for Buffer. Area under the curve (AUC) analysis demonstrated that ArtAB produced a significantly greater reduction in barrier resistance than either ArtB or Buffer (Figure 6C).

To investigate whether the reduction in paracellular resistance was associated with altered expression of tight junction (TJ) and TJ-associated genes, qRT-PCR was performed to evaluate occludin/ELL domain-containing protein 1 (*OCEL1*), E-cadherin (*CDH1*), claudin-1 (*CLDN1*), and claudin-3 (*CLDN3*) (Figure 6D–G). The 20 h time point was selected because ArtB- and Buffer-treated cells had begun to recover, whereas the resistance in ArtAB-treated cells continued to decline. *OCEL1*, *CDH1*, and *CLDN1* were significantly upregulated in ArtAB-treated cells relative to both ArtB and Buffer (Figure 6D–F). In contrast, *CLDN3* expression was significantly increased in ArtB-treated cells relative to Buffer, whereas ArtAB-treated cells exhibited a similar trend that did not reach statistical significance (Figure 6G).

## 3. Discussion

In this study, the cellular activity of purified ArtAB toxin and its binding subunit, ArtB, was assessed using a multi-omics approach in CHO-K1 cells. Equimolar concentrations of ArtAB and ArtB exhibited minimal cytotoxicity under the conditions tested. Proteomic analysis at 4 h revealed that ArtB elicited early cellular responses associated with intracellular trafficking and endolysosomal processing that were reduced following ArtAB treatment, while RNA-seq analysis demonstrated that ArtAB uniquely stimulated broad transcriptional remodeling. This included significant dysregulation of genes involved in cytoskeletal organization, which motivated subsequent evaluation of ArtAB and ArtB in C2BBe1 cells to assess epithelial barrier function. ArtAB produced a progressive reduction in barrier resistance, while ArtB did not elicit the same functional effect. Together, these findings establish a progression from molecular characterization to functional assessment across complementary cell models and identify molecular responses that can be investigated further in the context of ArtAB-induced epithelial barrier disruption.

The enrichment of GO terms associated with ER homeostasis, vesicular trafficking, and lysosomal processing among proteins more abundant following ArtB treatment suggests that the binding subunit alone elicits intracellular processing responses (Figure 2B,C). These biological functions are relevant to bacterial toxin uptake, as several AB_5_ toxins undergo endocytosis followed by retrograde trafficking through endosomal, Golgi, and ER compartments [34]. Representative proteins included the ER homeostasis factors HSPA5 and WFS1, the trafficking-associated proteins LMAN2 and TMEM167A, and the lysosomal enzyme HEXA (Figure 2D). Their increased abundance suggests that ArtB stimulates a host cell response and is therefore not biologically inert. Notably, ER stress responses involving HSPA5 have also been observed during *S.* Typhimurium infection [35]. However, the present study did not directly assess ArtAB and ArtB uptake, intracellular trafficking, or cellular localization, and differences in these processes may also contribute to the distinct molecular responses observed. Uniquely, these proteins were less abundant following ArtAB treatment relative to ArtB (Figure 2C and Table S1), suggesting that ArtAB may reduce cellular responses elicited by the binding subunit.

Notably, several redox-related proteins were less abundant in ArtAB-treated cells relative to ArtB-treated cells (Figure 2C). These proteins included the ER-resident proteins ERO1B and TMX1 (Table S1), which participate in disulfide-bond formation and protein folding [36,37,38]. Mitochondrial proteins associated with redox control and metabolism were also less abundant with ArtAB than with ArtB, including PRDX3, NDUFS1, and IDH3G (Table S1). PRDX3 functions as a mitochondrial antioxidant enzyme that directly detoxifies reactive oxygen species (ROS), while NDUFS1 and IDH3G contribute to electron transport and oxidative phosphorylation [39,40,41,42]. Similar alterations in host redox homeostasis and mitochondrial function occur during *Salmonella* infection [43,44]. The observed differences in PRDX3 and NDUFS1 are consistent with proteins implicated in ER stress and metabolic reprogramming [45,46]. However, direct assays measuring ROS or organelle function would be required to confirm these functional impacts. Together, these findings suggest that cellular responses associated with stress and redox homeostasis were less pronounced following ArtAB treatment relative to ArtB. Although replicate variability may have reduced statistical power in the ArtAB vs. Buffer comparison (Figure S2), the limited differential protein abundance at 4 h may also reflect a relatively modest early proteomic response to the holotoxin. Since only a single proteomic time point was evaluated, broader ArtAB-associated protein changes at later periods cannot be excluded.

In contrast to the limited early proteomic response to ArtAB, RNA-seq revealed broad early transcriptional remodeling following ArtAB treatment, whereas ArtB induced minimal transcriptional changes relative to Buffer (Figure 3). Hierarchical clustering of the final RNA-seq dataset further supported a distinct global transcriptional profile in ArtAB-treated cells relative to ArtB and Buffer (Figure S3). Downregulated genes in the ArtAB vs. Buffer and ArtAB vs. ArtB comparisons converged on pathways involved in growth and stress signaling, transcriptional regulation, and inflammatory signaling (Figure 3C). MAPK was among the most enriched shared pathways, featuring reduced expression of several activator protein-1- (AP-1) components, including *Fos*, *Fosb*, *Fosl1*, and *Junb* (Table S3) [47]. AP-1 integrates MAPK signaling with transcriptional programs controlling proliferation, stress response, and inflammation [48,49]. Expression of *Hbegf*, an upstream ligand that activates MAPK and lies within the erythroblastic leukemia viral oncogene homolog (ErbB) signaling pathway, was also reduced (Figure 3D) [50]. Supporting these pathway-level transcriptional changes, FOSL1 protein abundance was decreased by ArtAB (Table S1), and *Fos* downregulation was independently validated by qRT-PCR (Figure 3E). *Fos* is known to be an essential component of the AP-1 transcription complex that is involved in cell growth and proliferation [51]. Overall, these findings demonstrate a coordinated reduction in MAPK/AP-1 signaling following ArtAB treatment.

These shifts in growth signaling were accompanied by altered expression of cell-cycle regulators, including decreased *Plk2* and increased *Ccng2* (Figure 3D and Figure 5B), which are associated with growth inhibition and cell cycle arrest, respectively [52,53]. *Cdkn1a* mRNA expression and CDKN1A protein abundance were both reduced in ArtAB-treated cells (Table S3 and Figure 2D), providing cross-omics evidence of altered cell-cycle control [54]. Furthermore, decreased abundance of the cytokinesis-associated proteins ECT2 and RACGAP1 (Figure 2C and Table S2) points to significant disruption of host cell division processes [55]. Interestingly, ArtAB-treated cells exhibited significantly lower fluorescence than untreated cells at 12, 24, 48, and 72 h, indicating lower cytotoxicity at these later time points (Figure 1A,B). This finding could have been influenced by reduced cell proliferation, which may have limited the number of cells available to undergo membrane compromise and thereby contributed to the below-baseline fluorescence values observed with ArtAB. However, cell proliferation was not directly measured, and this possibility cannot be determined from the present cytotoxicity assay alone. With the minimal cytotoxicity observed, these findings demonstrate that ArtAB is associated with changes in cell-cycle regulatory networks in a manner consistent with reduced cell proliferation without triggering substantial cell death.

Beyond these effects on cell growth and cell-cycle regulation, a subset of downregulated genes in ArtAB-treated cells mapped to tumor necrosis factor (TNF), Janus kinase/signal transducer and activator of transcription (JAK-STAT), NF-κB, and IL-17 signaling (Figure 3C and Figure 4A), showing a potential coordinated alteration of several interconnected inflammatory pathways. These pathways included reduced expression of *Cxcl3*, *Il11*, and *Il6*, which contribute to inflammatory signaling and immune-cell recruitment (Figure 3D and Table S3). *Cxcl3* functions as a chemokine involved in neutrophil recruitment, whereas *Il6* and *Il11* are pleiotropic cytokines that regulate inflammation and tissue responses [56,57]. Reduced *Il6* expression was independently confirmed by qRT-PCR (Figure 4B). This inflammatory signature was further supported across both omics datasets, as *Icam1* gene expression was downregulated in the ArtAB vs. Buffer comparison (Figure 4A), and ICAM1 protein abundance was reduced in ArtAB relative to ArtB (Figure 2D). ICAM1 contributes to cell-surface adhesion and leukocyte interactions and is downstream of canonical inflammatory pathways [58]. Because CHO-K1 cells are not professional immune cells, these findings indicate an alteration of host cell-intrinsic inflammatory signaling rather than systemic immune suppression.

Cytoskeleton-associated transcriptional changes provided an additional link between the molecular response to ArtAB and its previously reported effects on CHO-K1 morphology. *Ppp1r12a* and *Rock2*, which regulate actomyosin organization and focal adhesion signaling, were downregulated (Figure 3D) [59,60]. The downregulation of these genes suggests a potential weakening of cell-substrate attachments, consistent with the altered cell rounding observed in CHO-K1 cells [27]. Conversely, TGF-β signaling was the only significantly enriched KEGG pathway among genes upregulated in ArtAB relative to ArtB (Figure 5A). TGF-β signaling regulates cellular processes, including growth, epithelial-to-mesenchymal transition (EMT), and cell-cell interactions [61]. Although KEGG enrichment does not prove functional activation of the entire pathway, ArtAB also increased the expression of *Snai1*, a key transcriptional regulator of EMT, which was validated by qRT-PCR (Figure 5B,C) [62]. Additional upregulated genes in both ArtAB comparisons included *Rhof* and *Arhgef15* (Figure 5B), further implicating Rho-family signaling and cytoskeletal regulation [63,64]. Together, changes in focal adhesion, TGF-β, and Rho-family signaling indicate that ArtAB modifies pathways controlling host cytoskeletal organization and cell-cell interactions.

DEGs downregulated following ArtAB treatment compared to Buffer were also enriched in the KEGG *Salmonella* infection pathway (Figure 4A). Genes mapped to this pathway reflected several recurring themes identified throughout our analysis, including intracellular trafficking, cell survival, inflammatory signaling, and cytoskeletal organization (Table S4). This convergence suggests that ArtAB influences host processes that are also targeted during intracellular infection, although pathway enrichment alone does not prove a direct role for ArtAB during bacterial pathogenesis. Collectively, these molecular responses, particularly those involving cytoskeletal organization and cell-cell connections, provided a rationale for directly evaluating epithelial barrier function. Using ECIS, we observed that ArtAB significantly reduced paracellular resistance (Ω) in C2BBe1 cells over 48 h compared to ArtB and Buffer (Figure 6B,C). Although all treatment groups initially exhibited a decrease in resistance, ArtB- and Buffer-treated cells began to recover after approximately 15–20 h, whereas resistance in ArtAB-treated cells continued to decline (Figure 6B). This divergent phenotype demonstrates a holotoxin-specific effect on epithelial barrier integrity, a key feature of NTS pathogenesis [65,66]. Barrier disruption was also recently demonstrated for *Salmonella* TT, which increases blood-brain barrier permeability through its catalytic CdtB subunit [22]. Although the direct cellular targets and mechanisms of ArtAB remain distinct, TT provides a precedent for barrier disruption by a *Salmonella* AB_5_-like toxin.

Tight and adherens junctions are critical structural regulators of epithelial integrity and paracellular permeability [67]. Despite the progressive decline in resistance, ArtAB increased the expression of junction-associated genes *OCEL1*, *CDH1*, and *CLDN1* at 20 h (Figure 6D–F). *CLDN3* exhibited a similar upward trend following ArtAB treatment, though it did not reach statistical significance, whereas ArtB significantly increased *CLDN3* expression relative to Buffer (Figure 6G). This discordance between reduced resistance and elevated junction gene expression may represent a compensatory transcriptional response to the loss of barrier integrity. A similar compensatory response has been reported in endothelial cells, where barrier disruption triggers an increased synthesis of the adherens junction protein VE-cadherin [68]. Since epithelial barrier integrity is heavily regulated through junctional protein localization and post-translational modification [69,70], future studies investigating these factors, such as phosphorylation, will be essential to elucidate the precise mechanisms and physiological relevance of ArtAB-induced barrier dysfunction.

Beyond these findings, several broader limitations of the present study should be acknowledged. The present study was conducted using in vitro cellular models and therefore does not reproduce the complexity of host physiology, immune-cell interactions, tissue architecture, or toxin exposure during infection. The physiological concentration of ArtAB during NTS infection also remains unknown and the 10 µg/mL concentration used here represents an experimentally defined in vitro exposure selected based on reproducible cellular responses observed in our previous work [27]. In addition, the transcriptomic and proteomic analyses captured a single early time point of 4 h, and the persistence of these molecular responses at later time points remains unknown. The molecular and functional analyses were also performed in different cellular models, which limits direct mechanistic integration across datasets. Future studies using intestinal organoids, co-culture systems, and in vivo models can extend these findings and determine how ArtAB-associated molecular responses influence intestinal barrier function. Nevertheless, these findings provide an important insight into the cellular activities of ArtAB and identify key pathways for further exploration.

## 4. Conclusions

Overall, this study demonstrates that the *Salmonella* AB_5_ toxin ArtAB functions as a potent modulator of host cell physiology without causing overt cytotoxicity. By integrating transcriptomic, proteomic, and functional assays, we show that ArtAB induces broad alterations in cell growth, inflammatory signaling, and cytoskeletal organization in CHO-K1 cells, with a separate reduction in epithelial barrier resistance in C2BBe1 cells. The molecular and functional findings were generated in distinct in vitro models, and their relationship during NTS infection remains to be determined. Together, these results broaden our understanding of ArtAB-associated host responses and provide a framework for further investigation of its role in NTS pathogenesis. Future studies elucidating the specific cellular targets of ArtAB and ArtB will be important for understanding their contributions to *Salmonella* infection in agriculturally important animals and for informing strategies to reduce transmission to humans within a One Health framework.

## 5. Materials and Methods

### 5.1. Recombinant Protein Production

Plasmids pMB006 (ArtAB) and pLC001 (ArtB) were previously cloned into endotoxin-free ClearColi BL21(DE3) competent cells (Lucigen, LGC Biosearch Technologies, Petaluma, CA, USA). ArtAB and ArtB were independently extracted and purified via fetuin agarose affinity chromatography as previously described (Figure S4) [27]. Fractions were collected in 1.0 mL aliquots and visualized with SDS-PAGE. Protein-containing fractions were dialyzed at 4 °C against 1× PBS + 5% glycerol using 12,000 Da MWCO regenerated cellulose dialysis tubing (Thermo Fisher Scientific, Waltham, MA, USA). One buffer change occurred after 12 h of dialysis. Following dialysis, proteins were concentrated with a Pierce 30K PES MWCO centrifugal filter (Thermo Fisher Scientific). Molecular weight and protein purity were evaluated with SDS-PAGE, and protein concentrations were determined using a Pierce Bicinchoninic Acid (BCA) Protein Assay Kit (Thermo Fisher Scientific).

### 5.2. Cell Culture and Maintenance

Chinese hamster ovary (CHO-K1, ATCC, Manassas, VA, USA; CCL-61) cells were cultured in Ham’s F-12 Nutrient Mix (Gibco, Thermo Fisher Scientific, Waltham, MA, USA) supplemented with 10% fetal bovine serum (FBS; Gibco, Thermo Fisher Scientific) and 1% penicillin/streptomycin (Thermo Fisher Scientific). C2BBe1 (Caco-2 clone, ATCC; CRL-2102) cells were cultured in Dulbecco’s Modified Eagle’s Medium (ATCC) with 10% FBS, 1× MEM non-essential amino acids (Gibco, Thermo Fisher Scientific), and 1% penicillin/streptomycin. Cells were maintained in tissue culture treated T75 flasks in a humidified incubator at 37 °C with 5% CO_2_. C2BBe1 cells were passaged at 80–90% confluency using 0.25% Trypsin-EDTA (Thermo Fisher Scientific), whereas CHO-K1 cells were passaged using 1× TrypLE Express Enzyme (Gibco, Thermo Fisher Scientific). For CHO-K1 starvation periods, cells were cultured in Ham’s F-12 Nutrient Mix supplemented only with 1% penicillin/streptomycin.

### 5.3. Cytotoxicity Assay

Cytotoxicity was measured using the CellTox Green Cytotoxicity Assay (Promega, Madison, WI, USA; #G8741) based on the manufacturer’s “Express, No-Step Addition at Dosing” protocol, with modifications described below. CHO-K1 cells were seeded into black-walled, clear-bottom, tissue culture-treated 96-well plates (Falcon, Corning, Corning, NY, USA; #353219) at 2500 cells per well in 200 µL of complete medium and incubated overnight. Before treatment, cells were starved in serum-free medium for 4 h. The serum-free medium was then replaced with equimolar concentrations of ArtAB (10 µg/mL) or ArtB (7.25 µg/mL), or an equivalent volume of Buffer (1× PBS + 5% glycerol). CellTox Green Dye was added at dosing according to the manufacturer’s protocol, and untreated cells containing medium only and cell-free background wells were included as controls.

Fluorescence measurements (490/525 nm) were collected from the same wells using a BioTek Cytation 3 Cell Imaging Multi-Mode Reader (BioTek Instruments, Winooski, VT, USA) with Gen5 software (version 2.07) at 4, 8, 12, 24, 48, and 72 h for continuous monitoring of cytotoxicity. A separate positive-control plate containing lysis solution-treated wells was used to generate maximum-positive controls at 4, 24, 48, and 72 h. Lysis solution was added 1 h prior to each fluorescence measurement rather than the manufacturer’s recommended 30 min incubation. Lysis solution dilutions were approximately 1:25 at 4 h, 1:20 at 24 h, and 1:15 at 48 and 72 h. Background fluorescence values from each cell-free well containing CellTox Green Dye and complete medium were averaged, and the mean background value was subtracted from all measurements. Three independent experiments were performed. Technical replicates were averaged at each time point within each experiment, and the resulting means were used for statistical analysis (*n* = 3 per treatment).

### 5.4. Apoptosis Assay

Apoptosis was measured using the Caspase-Glo 3/7 Assay (Promega; #G8090). CHO-K1 cells were seeded into solid white, tissue culture-treated 96-well plates (Falcon, Corning; #353296) at 2500 cells per well in 100 µL of complete medium and incubated overnight. Before treatment, cells were starved in serum-free medium for 4 h. Cells were then treated with 10 µg/mL ArtAB, 7.25 µg/mL ArtB, or an equivalent volume of Buffer. Separate plates were prepared for the 4 and 24 h treatment periods. Untreated cells containing medium only and cell-free background wells were included as controls. 

STS was prepared in DMSO and added at a final concentration of 5 µM 4 h before each luminescence measurement. Accordingly, STS was added at the time of treatment for the 4 h measurement and at 20 h for the 24 h measurement. At each endpoint, 100 µL of equilibrated Caspase-Glo 3/7 reagent was added to each well, and the plates were gently shaken to induce cell lysis. Reactions were incubated for 1 h at room temperature, protected from light, before luminescence was measured using a BioTek Cytation 3 Cell Imaging Multi-Mode Reader with Gen5 software. Background luminescence values from each cell-free well containing Caspase-Glo 3/7 reagent and complete medium were averaged, and the mean background value was subtracted from all measurements. One representative independent experiment was plotted and statistically analyzed for each time point, with technical replicate wells serving as the plotted observations (*n* = 4 per treatment).

### 5.5. Protein Extraction for Proteomics

CHO-K1 cells were seeded in 12-well tissue culture-treated plates at a density of 150,000 cells per well in 2 mL of complete medium. Before treatment, cells were starved in serum-free medium for 4 h. Cells were then treated with 10 µg/mL ArtAB, 7.25 µg/mL ArtB, or an equivalent volume of Buffer for 4 h. After treatment, cells were washed twice with ice-cold 1× PBS and lysed in RIPA lysis and extraction buffer (Thermo Fisher Scientific) supplemented with 1× HALT Protease and Phosphatase Cocktail (Thermo Fisher Scientific). Cell lysates were collected using a sterile cell scraper, pipetted into sterile microcentrifuge tubes, and stored at −80 °C overnight (*n* = 5 per treatment group). The samples were then thawed on ice and centrifuged at 14,000× *g* for 15 min to pellet cellular debris. The clarified supernatants were transferred to fresh tubes, and total protein concentrations were quantified with a BCA assay. Protein samples were shipped on dry ice to the IDeA National Resource for Quantitative Proteomics (Little Rock, AR, USA) for downstream proteomic analysis.

### 5.6. Mass Spectrometry Data Acquisition

The core facility performed sample preparation, including detergent removal and tryptic digestion, prior to mass spectrometry analysis. Desalted tryptic peptides in aqueous solution were injected from a 96-well plate. Up to 1 μg of each peptide sample was drawn into a 10 μL sample loop using a Dionex UltiMate 3000 Autosampler (Thermo Fisher Scientific) and loaded onto a 0.5 cm × 75 μm trapping column packed with Waters XSelect CSH C18 3.5 μm resin (Waters Corporation, Milford, MA, USA). Peptides were subsequently separated on a 15 cm × 75 μm analytical column packed with Waters XSelect CSH C18 2.5 μm resin (Waters Corporation) using an acetonitrile gradient over a 60 min data-independent acquisition (DIA) run.

Mass spectrometry was performed on an Orbitrap Exploris 480 Mass Spectrometer (Thermo Fisher Scientific). The instrument was configured to acquire a full MS1 precursor scan at a resolution of 60,000, followed by 50 consecutive DIA MS2 spectra using 12 *m*/*z* precursor isolation windows at a resolution of 15,000. A staggered window pattern with optimized window placements was employed for precursor isolation.

### 5.7. Proteomic Data Processing and Statistical Analysis

Raw DIA data were processed by the core facility using Spectronaut (version 20.1; Biognosys AG, Schlieren, Switzerland) in directDIA mode. Protein identification and quantification were performed against a UniProt *Cricetulus griseus* (Chinese hamster) protein database (release 2025_02; 23,887 protein entries). Protein inference was performed using the IDPicker algorithm, and precursor, peptide, and protein identifications were filtered using a target-decoy strategy with a false discovery rate (FDR) of 1% (*q* < 0.01). Data quality and normalization methods were evaluated using ProteiNorm [71]. Variance stabilizing normalization (VSN) was initially selected, and sample quality was further assessed by the proteomics core facility using principal component analysis (PCA), Pearson correlation analysis, and Euclidean distance analysis. Based on this QC assessment, ArtAB_3 and Buffer_5 were excluded prior to downstream statistical analysis. Following outlier removal, normalization methods were re-evaluated by the core facility using ProteiNorm, and cyclic loess normalization was selected for the final dataset. Log_2_-transformed, cyclic loess-normalized exclusive protein intensities were used for downstream analysis.

Differential protein abundance was assessed by the proteomics core facility using its in-house bioinformatics pipeline implemented with the proteoDA R package (version 2026.06.0+242), which applies limma linear models and empirical Bayes moderation [72]. Resulting *p*-values were adjusted using the Benjamini–Hochberg (BH) method, and proteins with an adjusted *p*-value (*p*_adj_) < 0.055 were considered differentially abundant. The final QC-filtered proteomics dataset was subsequently visualized in RStudio (version 2026.06.0+242) by PCA, Euclidean distance heatmap, and average Euclidean distance to each treatment group (Figure S2A,B).

### 5.8. Functional Enrichment Analysis of Proteomics Data

Differentially abundant proteins from the ArtAB vs. Buffer, ArtB vs. Buffer, and ArtB vs. ArtAB comparisons were manually curated prior to functional enrichment analysis. All UniProt identifiers in the core facility master list were reviewed using the UniProt ID Mapping tool following the UniProt 2026_02 update. Identifiers that no longer mapped to active UniProtKB entries and instead resolved to UniParc archive records were excluded from downstream analyses (Table S1). The remaining proteins were separated into higher- and lower-abundance groups for each comparison based on the differential abundance results provided by the proteomics core facility.

Gene symbols corresponding to differentially abundant proteins, supplied by the core facility, were analyzed using g:Profiler (version e114_eg62_p19_27110d83) with Chinese hamster CHOK1GS selected as the organism. The background consisted of all annotated proteins retained as active UniProtKB entries following the *Cricetulus griseus* proteome 2026_02 update. Gene Ontology molecular function (MF), cellular component (CC), biological process (BP), and KEGG pathway enrichment analyses were performed using a BH-adjusted *p*-value threshold of 0.05. Enrichment analysis was not performed for the ArtAB vs. Buffer comparison or the lower-abundance ArtB vs. Buffer dataset because of the limited number of differentially abundant proteins. The lower-abundance ArtB vs. ArtAB dataset was additionally evaluated using an exploratory threshold of 0.10 to increase sensitivity for functional enrichment analysis. For visualization, representative GO terms and KEGG pathways were selected based on statistical significance, the number of mapped proteins, biological relevance, and coverage of distinct functional themes. Complete enrichment outputs are provided in Table S2. Fold enrichment for the selected terms shown in Figure 2E was calculated using the equation:

$$Fold\;Enrichment=\frac{Intersection\text{\_}Size/Query\text{\_}Size}{Term\text{\_}Size/Effective\text{\_}Domain\text{\_}Size}$$

### 5.9. RNA Isolation, Library Preparation, and Sequencing

CHO-K1 cells were seeded, serum-starved, and treated with ArtAB, ArtB, or Buffer under the conditions described in Section 5.5. After treatment, cells were washed with 1× PBS and dissociated using 1× TrypLE Express Enzyme. Cells were pelleted at 200× *g* for 5 min. Total RNA was isolated using the GeneJET RNA Purification Kit (Thermo Fisher Scientific; #K0731) according to the manufacturer’s instructions and stored at −80 °C until further analysis (*n* = 4 per treatment group). RNA concentration was quantified using a NanoDrop spectrophotometer, and RNA integrity was evaluated by bleach agarose gel electrophoresis [73]. RNA samples were shipped on dry ice to Novogene (Berkeley, CA, USA).

At Novogene, RNA quantity, purity, and integrity were independently assessed with a NanoDrop spectrophotometer and Agilent 2100 Bioanalyzer (Agilent Technologies, Santa Clara, CA, USA) before poly(A)-enriched, non-strand-specific mRNA library preparation. Messenger RNA was isolated using poly-T oligo-attached magnetic beads, fragmented, and reverse-transcribed using random hexamer primers. Following second-strand cDNA synthesis, libraries were prepared by end repair, A-tailing, adaptor ligation, size selection, amplification, and purification. Library concentrations were measured by Qubit fluorometry and real-time PCR, and fragment-size distributions were analyzed using the Agilent 2100 Bioanalyzer. Libraries were then pooled and sequenced as 150 bp paired-end reads on an Illumina NovaSeq platform, targeting 6 Gb of raw sequence data per sample.

### 5.10. RNA-Seq Data Processing and Functional Enrichment Analysis

Initial bioinformatic processing and differential gene expression analysis were performed by Novogene using all biological replicates. Raw reads were processed using fastp to remove adaptor-containing, poly-N, and low-quality reads. Clean paired-end reads were aligned to the NCBI *Cricetulus griseus* reference genome assembly GCF_003668045.3 (CriGri-PICRH-1.0) using HISAT2 (version 2.2.1). Gene-level read counts were generated using featureCounts (version 2.0.6). Differential gene expression analysis was performed using the DESeq2 R package (version 1.42.0) for the ArtAB vs. Buffer, ArtAB vs. ArtB, and ArtB vs. Buffer comparisons.

Sample-level QC analyses performed by Novogene showed that one ArtB biological replicate, ArtB_2, separated from the remaining ArtB samples by PCA (Figure S5A). As an independent downstream QC assessment, Euclidean distance analysis was performed in RStudio and confirmed that ArtB_2 clustered more closely with ArtAB samples than with the remaining ArtB samples (Figure S5B). Based on these combined QC analyses, ArtB_2 was excluded, and the differential expression analyses for all three comparisons were repeated in NovoMagic using the remaining samples (Table S3). Raw *p*-values from the repeated analysis were adjusted for multiple testing using the BH method, and genes with a *p*_adj_ ≤ 0.05 were considered differentially expressed. Following ArtB_2 exclusion, the PCA generated in NovoMagic and Euclidean distance analysis performed in RStudio showed improved clustering of the remaining ArtB replicates (Figure S5C,D). DEGs from the final RNA-seq dataset were visualized using bidirectional hierarchical clustering of genes and samples to assess treatment-associated transcriptional profiles (Figure S3).

Differentially expressed genes (DEGs) from the final RNA-seq dataset were used for KEGG pathway enrichment analysis in NovoMagic using the clusterProfiler R package (version 4.8.1) with *Cricetulus griseus* (Chinese hamster; organism code cge). Pathways with a BH-adjusted *p*-value < 0.05 were considered significantly enriched (Table S4). For visualization, representative KEGG pathways were selected based on statistical significance, the number of mapped genes, biological relevance, and coverage of distinct functional themes.

### 5.11. qRT-PCR Analysis of CHO-K1 Cells

CHO-K1 cells were seeded, serum-starved, and treated with ArtAB, ArtB, or Buffer under the conditions described in Section 5.5. Total RNA was isolated, quantified, and evaluated for integrity as described in Section 5.9. To minimize amplification from genomic DNA (gDNA), RNA samples were treated with DNase I (Thermo Fisher Scientific) prior to reverse transcription. cDNA was synthesized using the RevertAid First Strand cDNA Synthesis Kit with Oligo(dT)_18_ primers (Thermo Fisher Scientific; #K1621) according to the manufacturer’s instructions (*n* = 4 per treatment group).

qPCR reactions were performed in MicroAmp Fast Optical 96-Well Reaction Plates (Applied Biosystems, Thermo Fisher Scientific, Waltham, MA, USA; #4346906). Each 20 µL reaction was prepared according to the manufacturer’s instructions using 2× SYBR Green qPCR Master Mix (Thermo Fisher Scientific; #A59529), gene-specific forward and reverse primers, 5–10 ng of cDNA template, and nuclease-free water. Primer pairs targeting *Fos*, *Il6*, and *Snai1* were designed using NCBI Primer-BLAST (Primer3 version 2.5.0), while primers for the reference gene *Gnb1* were based on a previously published sequence [74]. All primers were synthesized by Integrated DNA Technologies (IDT, Coralville, IA, USA), and the primer sequences are provided in Table S5. qPCR was performed using a StepOnePlus Real-Time PCR System (Applied Biosystems, Thermo Fisher Scientific) with StepOne Software (version 2.3) according to the 2× SYBR Green qPCR Master Mix manufacturer’s recommended cycling conditions. Relative gene expression was calculated using the ΔΔC_T_ method and reported as log_2_ fold change.

### 5.12. ECIS

Each well of an 8W10E+ array (Applied Biophysics, Troy, NY, USA) was pretreated with 200 µL of 10 mM L-Cysteine Monohydrate HCl (pH 7.4) for 10 min at room temperature. Arrays were washed twice with sterile water to remove residual L-cysteine. C2BBe1 cells were washed with 1× PBS + 0.5 mM EDTA and dissociated using 0.25% Trypsin-EDTA. After neutralization with complete medium, cells were centrifuged at 200× *g* for 5 min. Cell pellets were resuspended in 4 mL of complete medium, and 1 mL of the suspension was diluted into 7 mL of complete medium for ECIS seeding (1:8 dilution). A total volume of 400 µL was added to each well, and arrays were gently swirled to promote even seeding. Arrays were allowed to settle for 15 min at room temperature before overnight incubation.

On the following day, arrays were connected to an ECIS Z-Theta 16-Well Array Station (Applied Biophysics) using ECIS Software (version 1.215.0 PC). Multiple Frequency/Time (MFT) measurements were collected at 4000 Hz at 160 s intervals. After baseline measurements were established, a partial medium change of 200 µL per well was performed using equilibrated complete medium. Once stable resistance (~2000 Ω) and capacitance (~10 nF) values were reached, indicative of strong cell-cell connections (e.g., tight junctions), treatments were administered. ArtAB, ArtB, or an equivalent volume of Buffer were prepared as 40× stocks and added in 10 µL volumes to achieve final treatment concentrations of 10 µg/mL ArtAB and 7.25 µg/mL ArtB. Resistance (Ω) was monitored for 48 h after treatment and normalized within each well to the resistance measured immediately before treatment (*T*_0_ = 1). The area under the curve (AUC) below the baseline (*y* = 1) was calculated from all normalized resistance measurements over the 48 h treatment period. Three independent experiments were performed, and the AUC was calculated separately within each experiment. The resulting AUC values were combined for statistical analysis (*n* = 3 per treatment group).

### 5.13. qRT-PCR Analysis of C2BBe1 Cells

C2BBe1 cells were seeded in 12-well tissue culture-treated plates at a density of 65,000 cells per well in 2 mL of complete medium. Cells were cultured for 2 days to approximately 80% confluence. ArtAB, ArtB, or an equivalent volume of Buffer were prepared as 40× stocks and added in 50 µL volumes to achieve final treatment concentrations of 10 µg/mL ArtAB and 7.25 µg/mL ArtB. After 20 h, cells were washed with 1× PBS, and lysis buffer from the GeneJET RNA Purification Kit was added directly to each well. Cell lysates were collected using a sterile cell scraper, and total RNA was purified according to the manufacturer’s instructions and stored at −80 °C until further analysis (*n* = 4 per treatment group). DNase I treatment and cDNA synthesis were performed as described in Section 5.11.

qPCR reactions were performed in MicroAmp Fast Optical 96-Well Reaction Plates. For *OCEL1*, *CLDN1*, and *CDH1*, 25 µL reactions were prepared according to the manufacturer’s instructions using RT^2^ SYBR Green Fluor qPCR Mastermix (Qiagen, Hilden, Germany; #330513). Commercial Qiagen primer assays were used for the target genes and the reference gene *ACTB*, with 25 ng of cDNA template and nuclease-free water (Table S6). For *CLDN3*, reactions with *GAPDH* as the reference gene were performed using 2× SYBR Green qPCR Master Mix under the conditions described for the CHO-K1 qPCR assays in Section 5.11. Gene-specific primer sequences for *CLDN3* and *GAPDH* are provided in Table S6. qPCR was performed using a StepOnePlus Real-Time PCR System with StepOne Software. Qiagen assays were performed according to the manufacturer’s recommended cycling conditions, whereas *CLDN3* reactions were performed using the cycling conditions described in Section 5.11. Relative gene expression was calculated using the ΔΔC_T_ method and reported as log_2_ fold change.

### 5.14. Statistical Analysis

All statistical analyses and AUC calculations were completed using GraphPad Prism (version 11.0.2). Specific statistical tests used for each experiment are described in the corresponding figure legends. These included one-way or two-way ANOVA with appropriate multiple-comparisons tests.

## Data Availability

The mass spectrometry proteomics data have been deposited to the ProteomeXchange Consortium via the PRIDE [75] partner repository with the dataset identifier PXD082171. The RNA sequencing data have been deposited in the NCBI Gene Expression Omnibus (GEO) with the accession number GSE342183.

## Supplementary Materials

The following supporting information can be downloaded at: https://www.mdpi.com/article/10.3390/toxins18090397/s1, Tables S1–S4 are provided as separate Excel workbooks. Table S1: Processed proteomics dataset and updated UniProt annotations. Protein-level proteomic data are presented with original protein annotations and updated UniProt accession mapping. Sample-level abundance values are reported as log_2_-transformed, cyclic loess-normalized exclusive intensities. Differential abundance statistics are provided for ArtAB vs. Buffer, ArtB vs. Buffer, and ArtB vs. ArtAB, and include log fold change (logFC), lower and upper confidence interval bounds (CI.L and CI.R), average intensity, moderated *t*-statistic, *B*-statistic, *p*-value, and adjusted *p*-value. The workbook includes the “UniProt Mapping—All Entries” worksheet containing all protein entries and their UniProt annotation status and the “UniProt Mapping—Active Only” worksheet containing entries with at least one currently active UniProtKB accession. Table S2: Functional enrichment analysis of differentially abundant proteins. Complete GO and KEGG pathway enrichment results generated using g:Profiler are provided for proteins with increased abundance in ArtB vs. Buffer and ArtB vs. ArtAB, as well as those with decreased abundance in ArtB vs. ArtAB. Separate worksheets are provided for each comparison and direction. For the downregulated ArtB vs. ArtAB dataset, enrichment results obtained at the indicated BH-adjusted *p*-value thresholds are provided as separately labeled sections within the same worksheet. Each output includes enrichment source, term name and identifier, adjusted *p*-value, −log_10_-adjusted *p*-value, term and query sizes, intersection size, effective domain size, and the proteins contributing to each enriched term. Table S3: RNA sequencing differential expression results. Differentially expressed genes are presented with sample-level DESeq2-normalized counts and treatment-average normalized counts. Differential expression statistics, including log_2_ fold change, *p*-value, and *p*_adj_ are provided. Gene annotations include gene ID, gene name, chromosome, genomic start and end positions, strand, gene length, biotype, gene description, and gene family. The workbook contains separate worksheets for ArtAB vs. Buffer, ArtAB vs. ArtB, and ArtB vs. Buffer. Table S4: Significantly enriched KEGG pathways among differentially expressed genes. KEGG pathways with *p*_adj_ < 0.05 are provided for downregulated genes in ArtAB vs. Buffer and ArtAB vs. ArtB, as well as upregulated genes in ArtAB vs. ArtB. Separate worksheets are provided for each comparison and direction. Reported information includes KEGG pathway identifier and description, gene and background ratios (GeneRatio and BgRatio), *p*-value, *p*_adj_, contributing gene IDs and gene names, and the number of genes associated with each pathway. Supplemental Figures S1–S5 and Tables S5 and S6 are in a single PDF document. Figure S1: Volcano plot of differential gene expression in ArtB-treated CHO-K1 cells relative to Buffer. Figure S2: Proteomics sample-level QC following exclusion of ArtAB_3 and Buffer_5. Figure S3: Hierarchical clustering heatmap of RNA-seq gene expression profiles from CHO-K1 cells treated with ArtAB, ArtB, or Buffer. Figure S4: Representative SDS-PAGE images of purified and dialyzed ArtAB (left) and ArtB (right). Figure S5: RNA-seq sample-level QC and removal of ArtB_2. Table S5: CHO-K1 primer pair sequences for reference and target genes utilized in qRT-PCR. Table S6: C2BBe1 commercial primer assay information and gene-specific primer sequences utilized in qRT-PCR.

## Abbreviations

The following abbreviations are used in this manuscript:

AP-1	Activator protein-1
C2BBe1	Caco-2 brush border expression cell line clone 1
CHO-K1	Chinese hamster ovary clone K1
CT	Cholera toxin
DT104	Definitive type 104
ECIS	Electric cell–substrate impedance sensing
EMT	Epithelial-to-mesenchymal transition
FOSL1	Fos-related antigen 1
IL-17	Interleukin-17
MAPK	Mitogen-activated protein kinase
NF-κB	Nuclear factor kappa B
NTS	Non-typhoidal *Salmonella*
PT	Pertussis toxin
ROS	Reactive oxygen species
STS	Staurosporine
TGF-β	Transforming growth factor beta
TJ	Tight junction
TS	Typhoidal *Salmonella*
TT	Typhoid toxin
