# Supplementary material for: Multi-Omics and Functional Analysis of Salmonella ArtAB Toxin Activity Reveals Transcriptomic Remodeling and Epithelial Barrier Dysfunction
Source: Toxins (Basel). 2026 Sep 17;18(9):397. doi: 10.3390/toxins18090397 (PMC13611614; doi:10.3390/toxins18090397)
Supplement: Supplementary file 1 [file toxins-18-00397-s001.zip › Souza et al Supplemental Materials.pdf]

Supplementary: Multi-Omics and Functional Analysis of *Salmonella* ArtAB Toxin Activity Reveals Transcriptomic Remodeling and Epithelial Barrier Dysfunction

Noah M. Souza, Alexander O. Hale, Richard S. Beard Jr. and Juliette K. Tinker

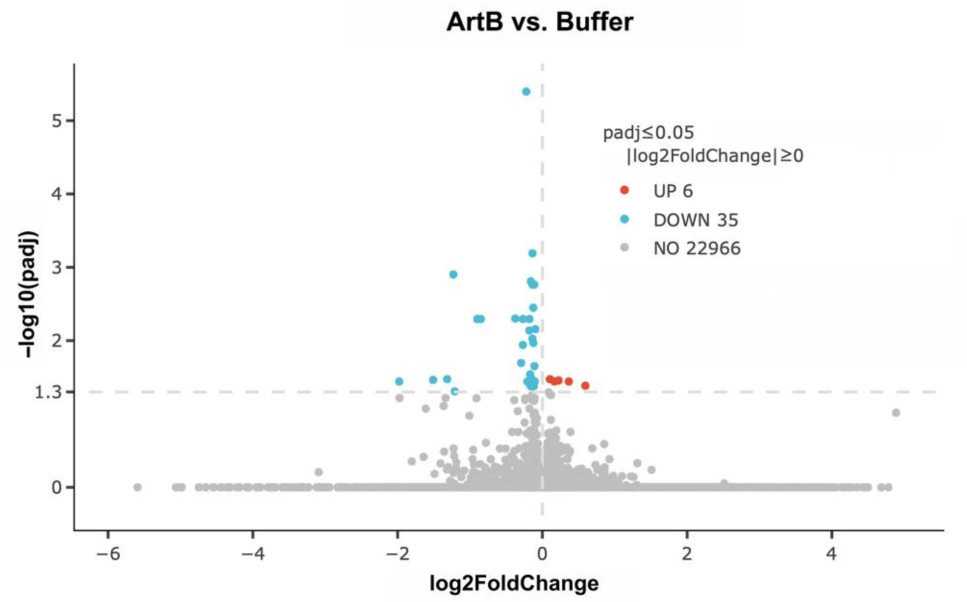

Figure S1. Volcano plot of differential gene expression in ArtB-treated CHO-K1 cells relative to Buffer. Cells were treated with 7.25 μg/mL ArtB or an equivalent volume of Buffer for 4 h.

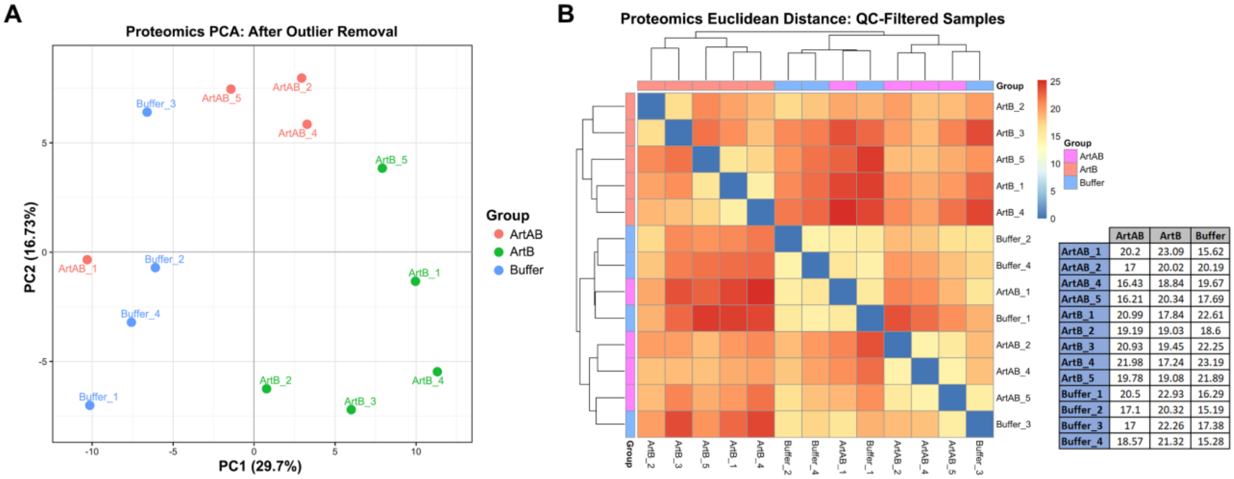

	ArtAB	ArtB	Buffer
ArtAB_1	20.2	23.09	15.62
ArtAB_2	17	20.02	20.19
ArtAB_4	16.43	18.84	19.67
ArtAB_5	16.21	20.34	17.69
ArtB_1	20.99	17.84	22.61
ArtB_2	19.19	19.03	18.6
ArtB_3	20.93	19.45	22.25
ArtB_4	21.98	17.24	23.19
ArtB_5	19.78	19.08	21.89
Buffer_1	20.5	22.93	16.29
Buffer_2	17.1	20.32	15.19
Buffer_3	17	22.26	17.38
Buffer_4	18.57	21.32	15.28

Figure S2. Proteomics sample-level QC following exclusion of ArtAB_3 and Buffer_5. All analyses were performed using $\log_2$-transformed, cyclic loess-normalized exclusive intensity values. (**A**) PCA of proteomics samples after core facility QC-based exclusion of ArtAB_3 and Buffer_5. (**B**) Euclidean distance heatmap after exclusion of ArtAB_3 and Buffer_5, with hierarchical clustering of samples based on Euclidean distance. Average Euclidean distances of each sample to each treatment group after ArtAB_3 and Buffer_5 exclusion are shown adjacent to the heatmap.

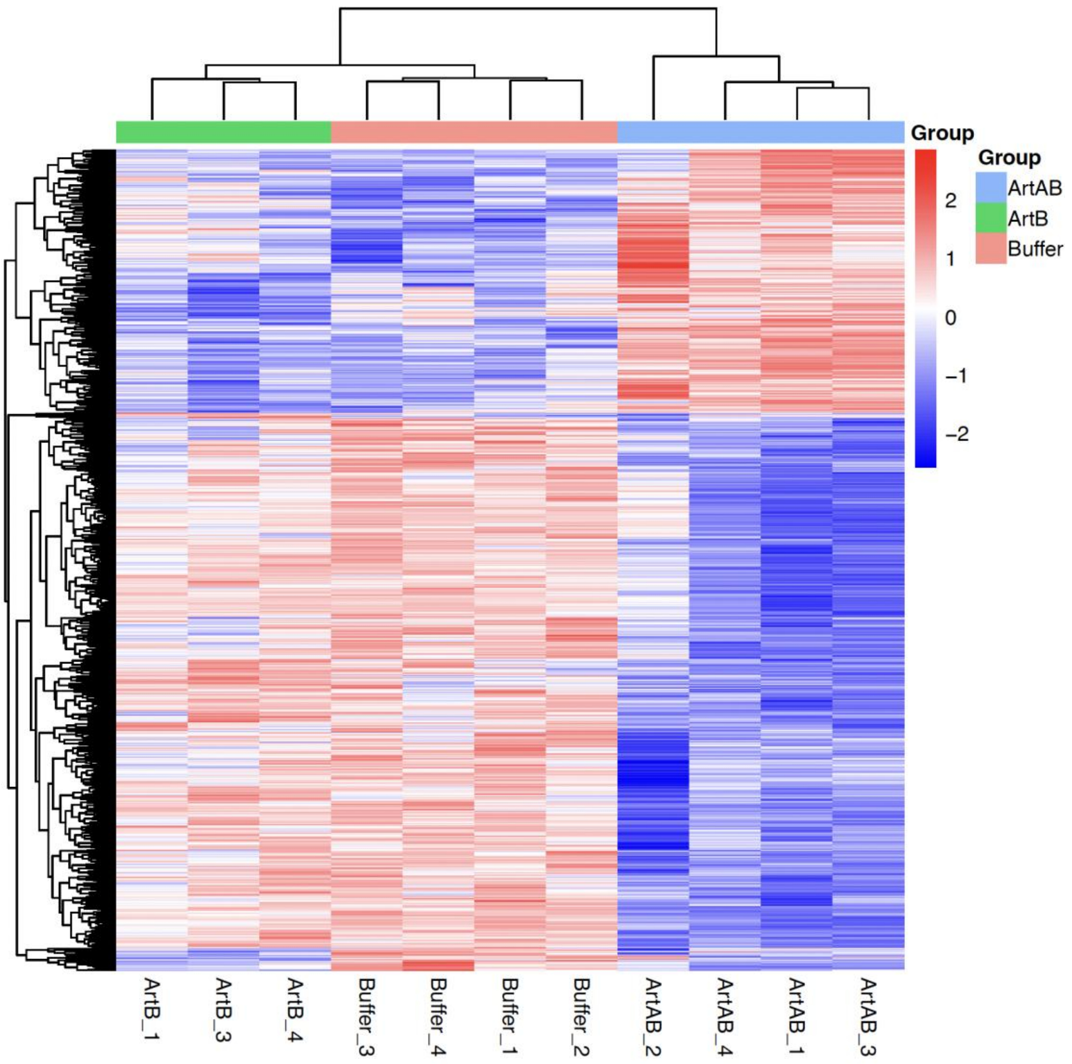

Figure S3. Hierarchical clustering heatmap of RNA-seq gene expression profiles from CHO-K1 cells treated with ArtAB, ArtB, or Buffer. CHO-K1 cells were treated with 10 μg/mL ArtAB, 7.25 μg/mL ArtB, or an equivalent volume of Buffer for 4 h. Differentially expressed genes (DEGs) from the final RNA-seq dataset, following exclusion of ArtB_2, were clustered across genes and samples using bidirectional hierarchical clustering. Red indicates relatively higher expression and blue indicates relatively lower expression across samples.

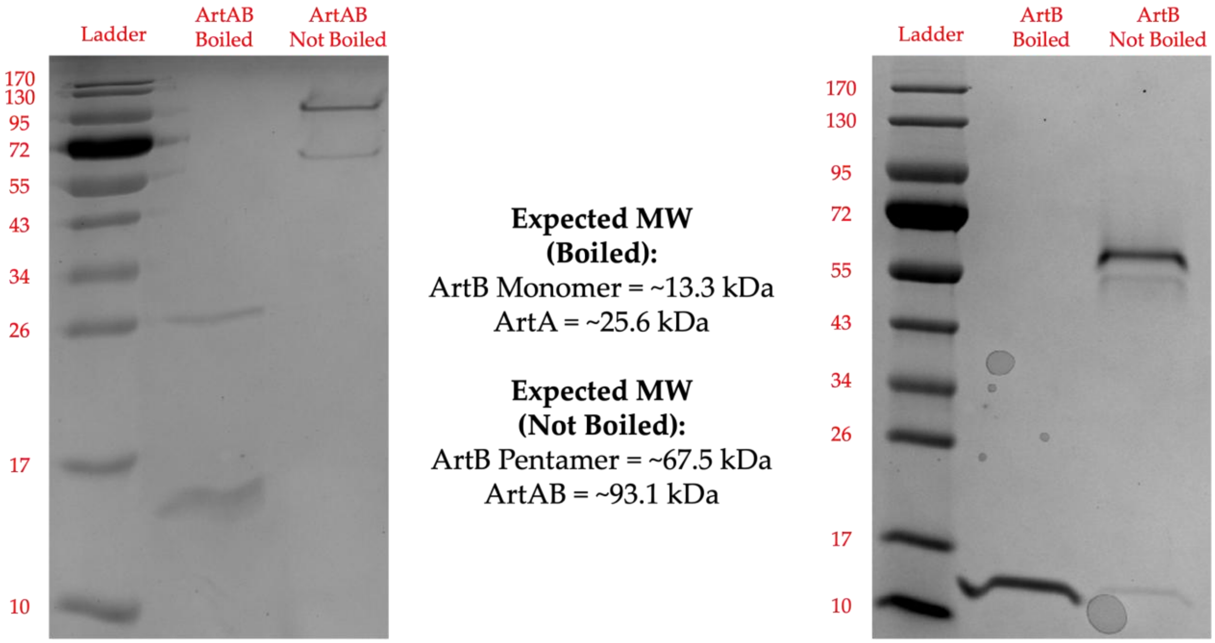

Figure S4. Representative SDS-PAGE images of purified and dialyzed ArtAB (left) and ArtB (right). ArtAB and ArtB samples were analyzed on separate gels under boiled (3 min) and non-boiled conditions; therefore, band intensities should not be directly compared between gels.

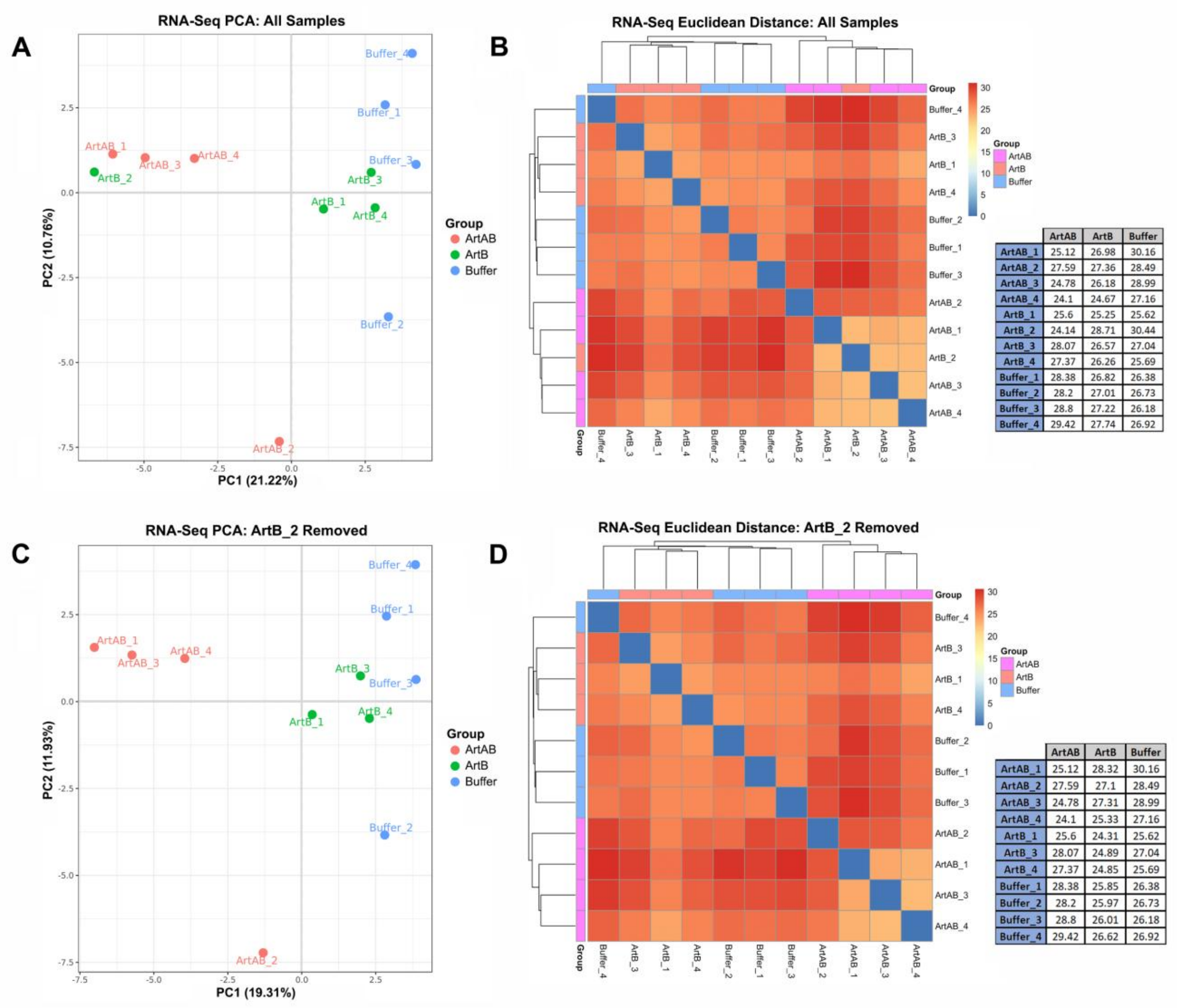

	ArtAB	ArtB	Buffer
ArtAB_1	25.12	26.98	30.16
ArtAB_2	27.59	27.36	28.49
ArtAB_3	24.78	26.18	28.99
ArtAB_4	24.1	24.67	27.16
ArtB_1	25.6	25.25	25.62
ArtB_2	24.14	28.71	30.44
ArtB_3	28.07	26.57	27.04
ArtB_4	27.37	26.26	25.69
Buffer_1	28.38	26.82	26.38
Buffer_2	28.2	27.01	26.73
Buffer_3	28.8	27.22	26.18
Buffer_4	29.42	27.74	26.92

	ArtAB	ArtB	Buffer
ArtAB_1	25.12	28.32	30.16
ArtAB_2	27.59	27.1	28.49
ArtAB_3	24.78	27.31	28.99
ArtAB_4	24.1	25.33	27.16
ArtB_1	25.6	24.31	25.62
ArtB_3	28.07	24.89	27.04
ArtB_4	27.37	24.85	25.69
Buffer_1	28.38	25.85	26.38
Buffer_2	28.2	25.97	26.73
Buffer_3	28.8	26.01	26.18
Buffer_4	29.42	26.62	26.92

Figure S5. RNA-seq sample-level QC and removal of ArtB_2. All analyses were performed using $\log_2$(FPKM + 1) expression values. (**A**) Principal component analysis (PCA) of all RNA-seq samples. (**B**) Euclidean distance heatmap of all RNA-seq samples, with hierarchical clustering of samples based on Euclidean distance. (**C**) PCA of remaining RNA-seq samples after removal of ArtB_2. (**D**) Euclidean distance heatmap after removal of ArtB_2, with hierarchical clustering of samples based on Euclidean distance. In panels B and D, the average Euclidean distances of each sample to each treatment group are shown adjacent to the heatmap.

Table S5. CHO-K1 primer pair sequences for reference and target genes utilized in qRT-PCR.

Gene Name & Symbol	Primer Pair Sequence	Source
G protein subunit beta 1 (*Gnb1*)	FW: CCATATGTTTCTTTCCCAATGGC<br>RV: AAGTCGTCGTACCCAGCAAG	Brown et al., 2018 [74]
Fos proto-oncogene (*Fos*)	FW: CTGTCAACGCACAGGACTTCT<br>RV: ACTGTAGGTCTGGGCTGGTG	This study (NCBI Primer-BLAST designed)
Interleukin-6 (*Il6*)	FW: GAGCCCACCAGGAACGAAA<br>RV: AGTGGATGGAAGTCTCTTGCG	This study (NCBI Primer-BLAST designed)
Snai1 family transcriptional repressor (*Snai1*)	FW: CGCACTCACACTGGTGAGAAA<br>RV: CAGCCAGACTCCTGGTGTTTA	This study (NCBI Primer-BLAST designed)

Table S6. C2BBe1 commercial primer assay information and gene-specific primer sequences utilized in qRT-PCR.

Gene Name & Symbol	Primer Information or Sequence	Source
Beta-actin (*ACTB*)	Commercial primer assay	Qiagen RT² qPCR Primer Assay (#PPH00073G-200)
Occludin/ELL domain-containing protein 1 (O*CEL1*)	Commercial primer assay	Qiagen RT² qPCR Primer Assay (#PPH19924A-200)
E-cadherin (*CDH1*)	Commercial primer assay	Qiagen RT² qPCR Primer Assay (#PPH00135F-200)
Claudin-1 (*CLDN1*)	Commercial primer assay	Qiagen RT² qPCR Primer Assay (#PPH02779A-200)
Glyceraldehyde 3-phosphate dehydrogenase (*GAPDH*)	FW: TGCACCACCAACTGCTTAGC<br>RV: GGCATGGACTGTGGTCATGAG	Laboratory primer (NCBI Primer-BLAST checked)
Claudin-3 (*CLDN3*)	FW: GCCTTCATCGGCAGCAACATCA<br>RV: AGCGAGTCGTACACCTTGCACT	Provided by Jorcyk Laboratory (NCBI Primer-BLAST checked)
